# The Probiotic *Parabacteroides johnsonii* Ameliorates Metabolic Disorders Through Promoting BCAAs to BSCFAs Conversion

**DOI:** 10.1002/advs.202502624

**Published:** 2025-08-07

**Authors:** Yimeng Chen, Shujun Jiang, Haolu Wang, Mengzhen Si, Haixia Wu, Xinyi Liang, Shenmeng Yao, Yanliang Zhang, Xiaodong Wen, Jie Yang

**Affiliations:** ^1^ Pukou Hospital of Chinese Medicine affiliated to China Pharmaceutical University China Pharmaceutical University 639 Longmian road Nanjing China; ^2^ State Key Laboratory of Natural Medicines School of Traditional Chinese Pharmacy China Pharmaceutical University 639 Longmian road Nanjing China; ^3^ Department of Infectious Diseases Nanjing Hospital of Chinese Medicine Affiliated to Nanjing University of Chinese Medicine Nanjing China

**Keywords:** BCAAs, BSCFAs, FGF1, metabolic disorders, parabacteroides johnsonii

## Abstract

The gut bacterium *Parabacteroides* spp. is increasingly recognized for its therapeutic potential in treating metabolic disorders. However, the role of *Parabacteroides johnsonii* in metabolic disorders is never reported. Here, this study finds that the abundance of *P. johnsonii* in the feces is negatively correlated with the blood glucose and lipid levels of obese patients. Oral administration of live *P. johnsonii* improved the metabolic dysfunction in high fat diet (HFD)‐fed mice, accompanied by the alleviation of leaky gut and the systemic inflammation. *P. johnsonii* enhanced the catabolism of branched‐chain amino acids (BCAAs) to branched‐chain short‐chain fatty acids (BSCFAs) in gut. Particularly, the conversion of valine to isobutyrate is correlated to the symptoms of obese patients. Isobutyrate intervention mirrored the favorable effects of *P. johnsonii* on HFD‐fed mice. Isobutyrate increased H3K14 acetylation at *Fgf1b* promoters and activated its transcription through inhibition of HDAC3 in colon, thereby maintaining the intestinal barrier integrity. The natural product stachyose exerted anti‐obesity effects by enhancing the growth of *P. johnsonii*. These results offered insights into the mechanisms underlying the potential of *P. johnsonii*, isobutyrate, and stachyose in treating metabolic disorders.

## Graphical abstract

Clinical discovery and the proposed underlying mechanism by how *P. johnsonii* ameliorated metabolic disorders.

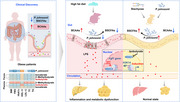

## 1 Introduction

The relationship between gut microbiota and human health has garnered extensive attention, particularly in the context of obesity. During the development of obesity, significant dysregulation occurs within the gut microbiota. This is principally featured by a decline in the number of probiotic bacteria and a concurrent rise in the population of harmful bacteria. The dysbiotic microbiota can initiate a cascade of detrimental effects. For instance, it impairs intestinal permeability. As a result, proinflammatory antigens, metabolites, and microbes can enter the bloodstream, triggering systemic chronic inflammation. It is well known chronic inflammation is an important driver of glucose and lipid disorders in the progression of obesity. Some probiotics, such as *Bifidobacterium* and *Lactobacillus*,^[^ ^1^, ^2^ ^]^ can preserve the normal state of the intestinal barrier and mitigate inflammatory responses thereby alleviating the dysfunction of glucose and lipid metabolism in obesity. Therefore, based on the regulatory role of probiotics, therapeutic strategies that focus on the gut microbiota may offer a novel approach for the prevention and treatment of obesity.

Branched – chain amino acids (BCAAs), namely leucine, isoleucine, and valine, are indispensable amino acids. They are crucial for protein synthesis and maintaining glucose balance via nutrient – mediated signaling pathways.^[^ ^3^ ^]^ However, studies have revealed that levels of BCAAs in plasma were increased in individuals with obesity and insulin resistance.^[^ ^4^ ^]^ With the widespread application of metabolome, increased levels of BCAAs are considered to be a metabolic hallmark of obesity and type 2 diabetes mellitus.^[^ ^5^, ^6^ ^]^ Excessive BCAAs could activate mammalian target of rapamycin complex 1 (mTORC1), oxidative stress and apoptosis of β cell leading to insulin resistant.^[^ ^4^ ^]^ Besides, excessive BCAAs can disrupt the metabolic balance of intestinal epithelial cells inducing intestinal inflammation.^[^ ^7^, ^8^ ^]^ Gut microbiota may modulate host BCAAs availability through directly utilize or metabolize BCAAs in the intestine.^[^ ^9^ ^]^ The latest research found that *Clostridium sporogenes* isolated from human feces could mediate the degradation of BCAAs into branched chain short chain fatty acids (BSCFAs) by *porA* gene.^[^ ^10^ ^]^ *Parabacteroides merdae* also could enhance intestinal BCAAs degradation and protect against cardiovascular damage.^[^ ^11^ ^]^ Thus, targeting gut microbiota driven‐BCAA catabolism is a potential strategy to attenuate obesity and its complications.

BSCFAs consisting of isobutyrate, 2‐methylbutyrate, and isovalerate, are produced through the fermentation of BCAAs. Just as acetate, propionate, and butyrate, BSCFAs are present in high concentrations within the colon.^[^ ^12^ ^]^ This abundance in the colon indicates their potential significance in the complex physiological processes occurring within the intestinal environment, where they may interact with the gut microbiota and host cells, potentially influencing various aspects of metabolism and overall health. While the functions of BCAAs and short chain fatty acids (SCFAs) in obesity have been well – studied, the relationship between BSCFAs and obesity remains little known. As we want to enhance the degradation of BCAAs to BSCFAs, it is essential to uncover the physiological roles of BSCFAs in obesity.

As a result of this study, we detected that the abundance of *Parabacteroides johnsonii* and the level of isobutyrate were significantly changed in obese patients. Supplementation of  live *P. johnsonii* could improve the metabolic symptoms of obese mice by metabolizing BCAAs into BSCFAs in the intestine. Isobutyrate, as one of BSCFAs, had similar effects as *P. johnsonii*. It promoted the expression of fibroblast growth factor 1 (FGF1) via inhibiting histone deacetylase 3 (HDAC3) activity, and consequently restoring intestinal barrier function and attenuating inflammation in metabolic tissues. In addition, we found that stachyose, a naturally occurring tetrasaccharide in various plants and traditional Chinese medicines, could promote the growth of *P. johnsonii* and improve glucose and lipid metabolism. Thus, this study highlighted the potential of *P. johnsonii* and isobutyrate as protective agents against obesity and provided insight into the underlying molecular mechanisms.

## 2 Results

### 2.1 Analysis of Gut Microbiota in Individuals with Obesity

**Figure 1:**
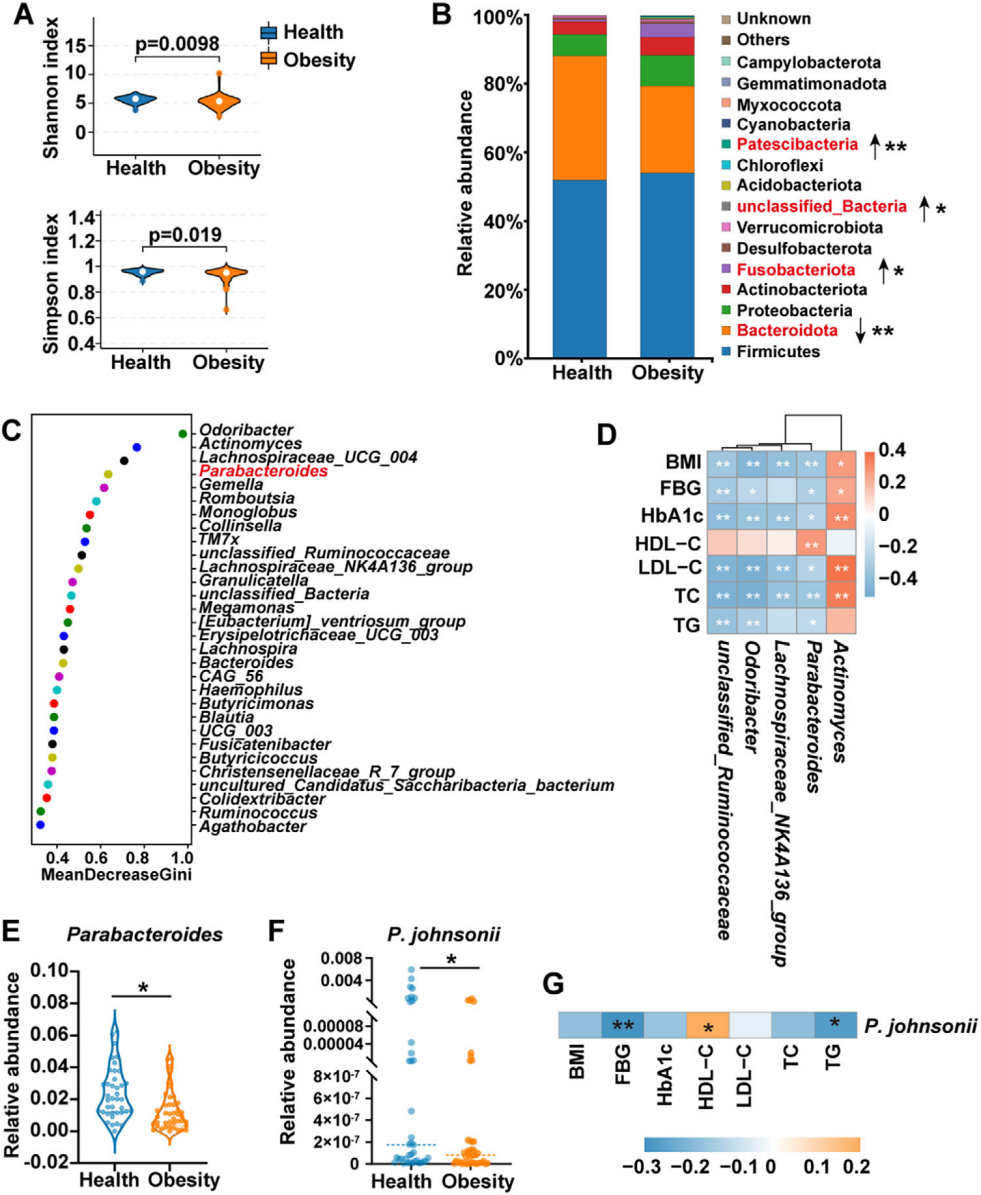
Gut microbiota profiling in obese patients. A) Alpha diversity analysis. B) Relative abundance of bacteria at the phylum level (percentage of total bacteria). Upwards arrow means increased in obese patients, and down arrow means decreased in obese patients. C) Random forest analysis at genus level. D) Spearman's analysis of the correlations between the relative abundance of microbiota and blood biochemical indexes in patients. E) Relative abundance of *Parabacteroides*. F) Relative abundance of *P. johnsonii* detected by QPCR. G) Spearman's analysis of the correlations between the relative abundance of *P. johnsonii* and blood biochemical indexes in patients. Health, healthy subjects (n = 39); Obesity, obese patients (n = 42); (B, E, F) values are means ± SD, (A, B, E, F) analyzed by Mann‐Whitney test. ^*^ *p* < 0.05, ^**^ *p* < 0.01, Health versus Obesity.

In order to delve into the connection between gut microbiome alterations and obesity, we contrasted the microbiota compositions of 42 obese patients with those of 39 healthy volunteers (Table S1, Supporting Information). The rarefaction curve indicated the volume of sequencing data was adequate to represent the species diversity in the samples (Figure S1A, Supporting Information). Although there was no significant difference in the species richness of gut microbiota between obese patients and healthy volunteers based on alpha diversity analysis (Figure S1B, Supporting Information), the microbiota diversity was significantly decreased in obesity (**Figure** **1**A). At the phylum level, the abundances of *Patescibacteria* and *Fusobacteriota* were significantly increased in obese patients, while the relative abundance of *Bacteroidota* was significantly decreased (Figure 1B). At the genus level, the top 30 important markers were obtained in descending order of importance through the random forest analysis (Figure 1C). Importantly, *Parabacteroides*, *Actinomyces*, *Odoribacter*, *Lachnospiraceae_NK4A136_group* and *unclassified_Ruminococcaceae* were linked with blood glucose and lipid levels (Figure 1D). Among them, we noticed that *Parabacteroides* was significantly decreased in obesity (Figure 1E) and it was negatively correlated with body mass index (BMI), fasting blood glucose (FBG), hemoglobin A1c (HbA1c), low‐density lipoprotein cholesterol (LDL‐C), total cholesterol (TC) and triglycerides (TG) levels but positively correlated with HDL‐C (Figure 1D). Furthermore, we found three species (*P. distasonis*, *P. goldsteinii*, *P. johnsonii*) in *Parabacteroides* were significantly decreased (Figure 1F; Figure S1C–E, Supporting Information) in obese patients. Notably, the relative abundances of *P. johnsonii* and *P. distasonis* were negatively correlated with blood glucose and lipid levels of the patients (Figure 1G; Figure S1F, Supporting Information), indicating the dysfunctions of *P. johnsonii* and *P. distasonis* might be involved in the pathological progression of obesity. It has been demonstrated that *P. distasonis* could act as a probiotic for regulating the metabolism of obese mice.^[^ ^13^, ^14^, ^15^ ^]^ Nevertheless, the function of *P. johnsonii* in obesity remains unclear. Hence, we focused on exploring the functions of *P. johnsonii* in obesity in the following study.

### 2.2 Live *P. johnsonii* (LPJ) Ameliorated Metabolic Disorders in HFD‐fed Mice

**Figure 2:**
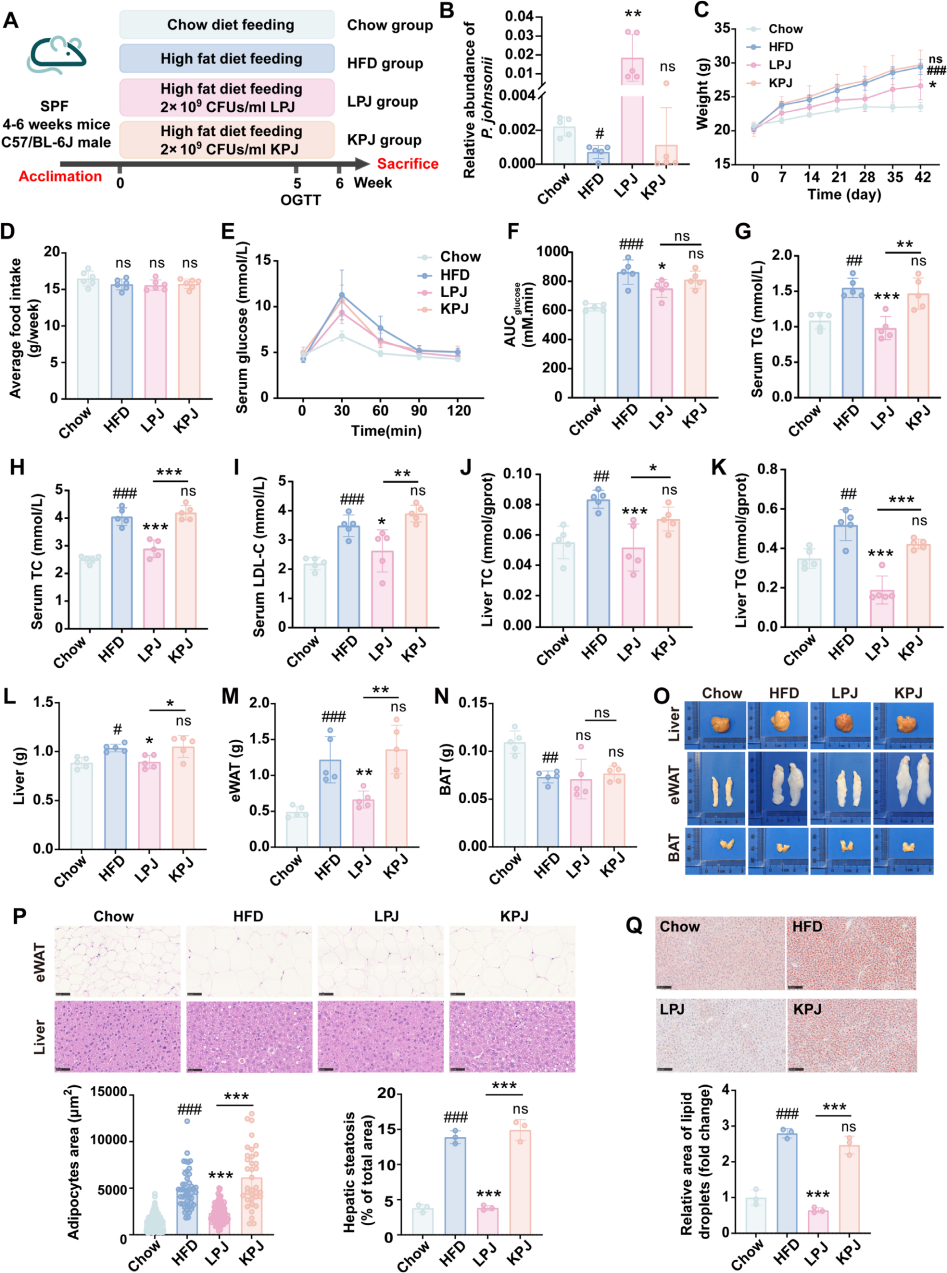
Oral administration of LPJ improved glucose and lipid metabolism disorders in HFD‐fed mice. A) Scheme of the experimental design. B) Relative abundance of LPJ. C) Body weight change curve. D) Cumulative food intake (n = 6). E) Oral glucose tolerance test. F) Area under the curve of OGTT. G‐I) The concentration of TG (G), TC (H), and LDL‐C (I) in serum. J,K) The level of TC (J) and TG (K) in liver. L‐N) The weight of liver (L), eWAT (M) and BAT (N). O) Representative images of tissue. P) Representative images of H&E in liver and eWAT (3 mice per group). Scale bars, 50 µm. Q) Representative images of Oil red O staining in liver (3 mice per group). Scale bars, 100 µm. Chow, chow diet‐fed mice; HFD, high fat diet‐fed mice; LPJ, live *P. johnsonii* treated mice; KPJ, heat‐killed *P. johnsonii* treated mice (n = 5). Data are presented as the means ± SD, analyzed by one‐way ANOVA for 3 or more groups and unpaired t test was used for two groups. Chow versus HFD, ^#^ *p* < 0.05, ^##^ *p* < 0.01, ^###^ *p* < 0.001; LPJ or KPJ versus HFD, ^*^ *p* < 0.05, ^**^ *p* < 0.01, ^***^ *p* < 0.001; ns, no significant difference.

Consistent with the results of obese patients, *P. johnsonii* was significantly decreased in HFD‐fed mice (**Figure** **2**A,B). Oral administration of LPJ instead of heat‐killed *P. johnsonii* (KPJ) could significantly reduce the weight gain induced by HFD (Figure 2C), whereas there was no significant difference in the food intake of mice (Figure 2D). While FBG levels were unchanged in HFD‐fed mice, HFD impaired oral glucose tolerance. LPJ treatment mitigated this impairment (Figure 2E,F). Meanwhile, LPJ intervention reduced the levels of serum TG (Figure 2G), TC (Figure 2H), and LDL‐C (Figure 2I). It also significantly down‐regulated the levels of TC and TG in liver (Figure 2J,K). The weights of liver and epididymal white fat tissue (eWAT) except brown fat tissue (BAT) were decreased by LPJ (Figure 2L–O). The results of H&E staining (Figure 2P) and Oil Red O staining (Figure 2Q) further demonstrated that LPJ could improve the size of adipocytes and lipid accumulation in the liver. We found HFD mice treated with KPJ did not show improvements in these parameters measured (Figure 2), indicating that cellular components made few contributions to the metabolic benefits of *P. johnsonii*.

Analysis of fecal excretion and jejunal lipid metabolism revealed that LPJ had no effect on fecal excretion volume, lipid absorption and transport genes *(Cd36, Fatp4*), TG synthesis genes (*Dgat, Lpcat3)*, and chylomicron synthesis gene (*Apob48*) (Figure S2, Supporting Information). However, LPJ treatment inhibited hepatic lipid uptake related gene expressions (*Fabp1* and *Fatp2*) and promoted lipid *β*‐oxidation related gene expressions (*Pgc1α* and *Cpt1α*) (Figure S3, Supporting Information). Therefore, the beneficial effects of *P. johnsonii* on obesity mainly related to its action on hepatic lipid uptake and *β*‐oxidation (Figure S3, Supporting Information).

In addition, the effects of LPJ on normal chow diet‐fed mice were also investigated (Figure S4A, Supporting Information). Although LPJ did not affect the body weight (Figure S4B, Supporting Information), food intake (Figure S4C, Supporting Information), and the weights of the liver, eWAT, and BAT (Figure S4D–F, Supporting Information) of mice, it could regulate glucose tolerance of mice (Figure S4G,H, Supporting Information), and reduce the levels of TG (Figure S4I, Supporting Information), TC (Figure S4J, Supporting Information), and LDL‐C (Figure S4K, Supporting Information) in serum.

These findings confirmed the protective effects of the gut symbiont *P. johnsonii* on metabolic disorders, thus indicating the potential of *P. johnsonii* as a good candidate for probiotic of ameliorating obesity and its related diseases.

### 2.3 LPJ Alleviated Inflammation and Improved Intestinal Mucosal Barrier Function in HFD‐fed Mice

**Figure 3:**
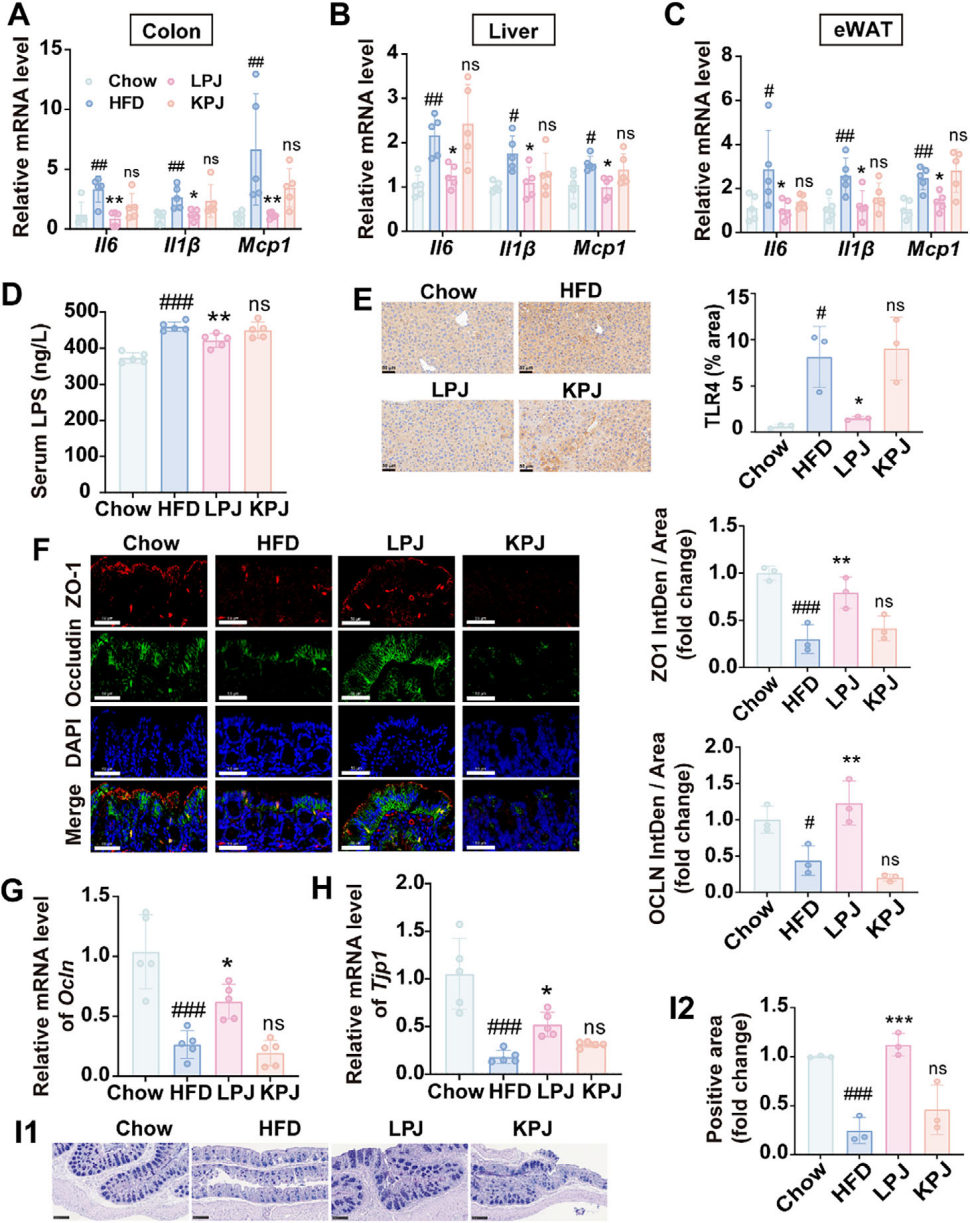
Oral administration of LPJ improved inflammation and intestinal mucosal barrier function in HFD‐fed mice. A‐C) Relative mRNA levels of inflammatory factors in colon (A), liver (B) and eWAT (C). D) The concentration of LPS in serum. E) Immunohistochemical staining of TLR4 in liver (3 mice per group). Scale bars, 50 µm. F) Intestinal immunofluorescence of ZO‐1 and Occludin (3 mice per group). Scale bars, 50 µm. G) Relative mRNA level of *Ocln* in colon. H) Relative mRNA level of *Tjp1* in colon. I) AB‐PAS staining of colon (3 mice per group). Scale bars, 100 µm. Chow, chow diet‐fed mice; HFD, high fat diet‐fed mice; LPJ, live *P. johnsonii* treated mice; KPJ, killed *P. johnsonii* treated mice (n = 5). Data are presented as the means ± SD, analyzed by one‐way ANOVA. Chow versus HFD, ^#^ *p* < 0.05, ^##^ *p* < 0.01, ^###^ *p* < 0.001; LPJ or KPJ versus HFD, ^*^ *p* < 0.05, ^**^ *p* < 0.01, ^***^ *p* < 0.001, ns, no significant difference.

Obesity is characterized by chronic inflammation in multiple organs.^[^ ^16^ ^]^ In our study, the mRNA expressions of pro‐inflammatory cytokines were measured in HFD‐fed mice with or without LPJ supplementation. It was found *Il6*, *Il1β* and *Mcp1* mRNA levels were significantly increased in colon (**Figure** **3**A), liver (Figure 3B) and eWAT (Figure 3C) of HFD‐fed mice but these trends were reversed by LPJ treatment, indicating LPJ could alleviate inflammation in HFD‐fed mice. KPJ had no influence on the mRNA expressions of these cytokines (Figure 3A–C). Consistent with these results, F4/80 expression was increased in the liver of HFD‐mice, indicating macrophage infiltration was induced by HFD. LPJ intervention alleviated the macrophage infiltration in liver (Figure S5, Supporting Information).

The disruption of intestinal permeability can cause lipopolysaccharide (LPS) to translocate into other tissues via the circulatory system. Once in the circulation, LPS can be recognized by toll – like receptors (TLRs) or other pattern – recognition receptors. This recognition event then triggers a cascade of events that lead to systemic inflammation.^[^ ^17^ ^]^ We found LPJ supplementation could down‐regulate the level of LPS in serum (Figure 3D) and TLR4 protein expression in the liver of HFD‐fed mice (Figure 3E). Furthermore, the mRNA and protein expressions of Occludin and ZO‐1 were suppressed in HFD‐fed mice, but were restored by LPJ (Figure 3F–H). Similarly, the AB‐PAS staining showed that HFD reduced mucin in the intestine, and LPJ intervention could increase the number of goblet cells (Figure 3I). These findings suggested the anti‐inflammation activity of *P. johnsonii* might be related to the remodeling of intestinal barrier function.

### 2.4 The Protective Effects of *P. johnsonii* on Antibiotics (ABX) Treated HFD‐fed Mice

**Figure 4:**
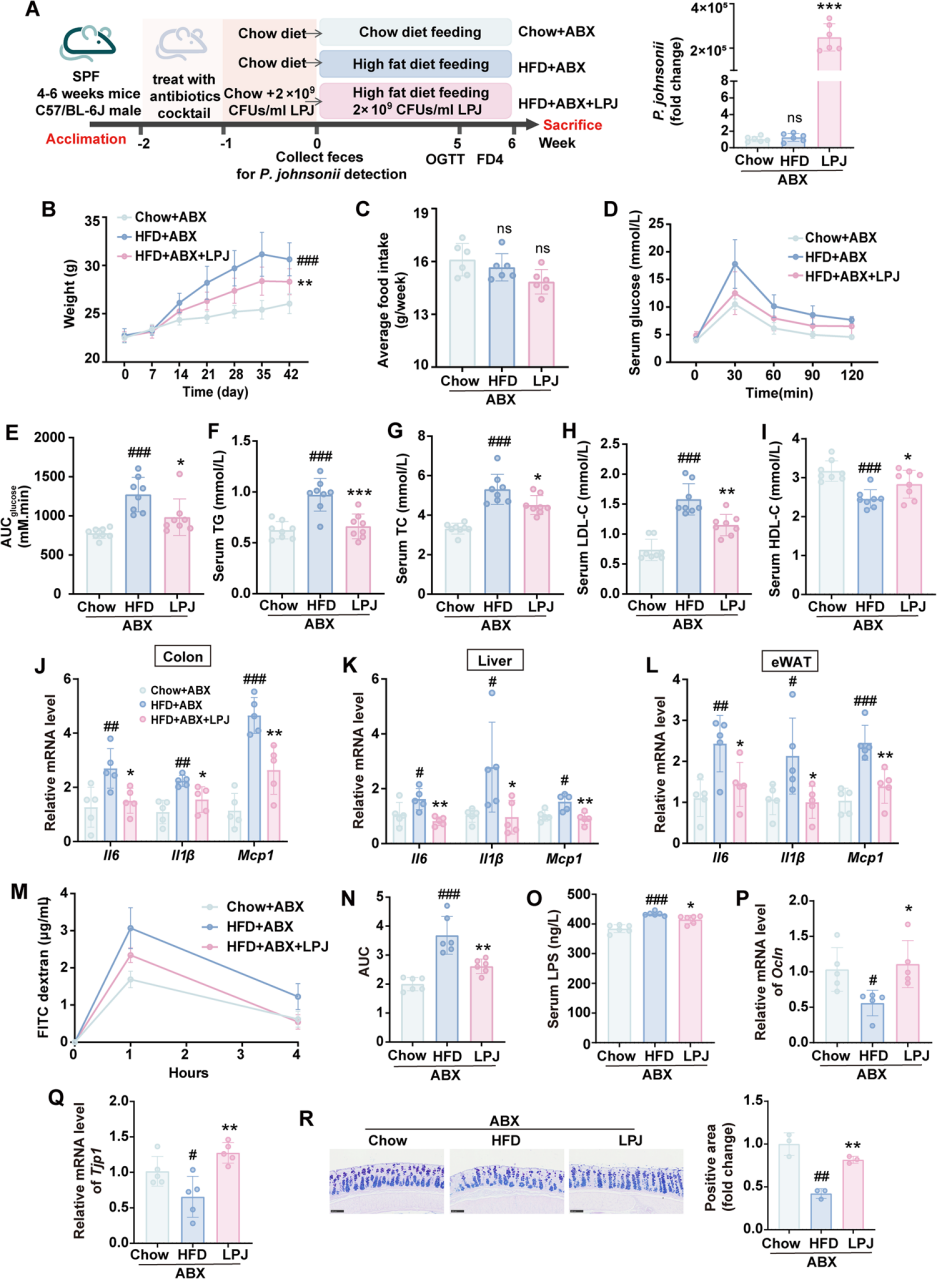
Oral administration of LPJ alleviated metabolic disorders and gut leak in HFD‐fed pseudo‐germ‐free mice. A) Scheme of the experimental design and the relative abundance of *P. johnsonii* before HFD fed. B) Body weight change curve. C) Cumulative food intake. D) Oral glucose tolerance test. E) Area under the curve of OGTT. F‐I) The concentration of TG (F), TC (G), LDL‐C (H) and HDL‐C (I) in serum. J‐L) Relative mRNA level of inflammatory factor in colon (J), liver (K) and eWAT (L). M) Intestinal FITC‐dextran test. N) Area under the curve of FITC‐dextran. O) The concentration of LPS in serum. P) Relative mRNA level of *Ocln* in colon. Q) Relative mRNA level of *Tjp1* in colon. R) AB‐PAS staining of colon (3 mice per group). Scale bars, 100 µm. ABX, antibiotics; Chow, chow diet‐fed mice; HFD, high fat diet‐fed mice; LPJ, live *P. johnsonii* (n = 5–8). Data are presented as the means ± SD, analyzed by one‐way ANOVA. Chow+ABX versus HFD+ABX, ^#^ *p* < 0.05, ^##^ *p* < 0.01, ^###^ *p* < 0.001; HFD+ABX+LPJ versus HFD+ABX, ^*^ *p* < 0.05, ^**^ *p* < 0.01, ^***^ *p* < 0.001, ns, no significant difference.

To enhance the colonization efficacy of *P. johnsonii*, we used ABX to deplete the gut microbes in mice (**Figure** **4**A). As expected, the abundance of *P. johnsonii* was dramatically increased after administered with LPJ in ABX‐treated mice (Figure 4A). Consistent with results in conventional mice model, LPJ could significantly decrease the body weight and improve metabolic disorders caused by HFD after ABX intervention (Figure 4B–I). Furthermore, LPJ also alleviated the hepatic lipid metabolism which was impaired by HFD (Figure S6, Supporting Information). The mRNA expressions of inflammatory factors (*Il6*, *Il1β* and *Mcp1*) were significantly lower in the colon, liver and eWAT of HFD+ABX+LPJ group compared with these in HFD+ABX group (Figure 4J–L).

In addition, oral administration of LPJ decreased the levels of FITC‐dextran and LPS in the serum in HFD+ABX‐fed mice (Figure 4M–O). *Tjp1* and *Ocln* mRNA expressions (Figure 4P,Q) and the secretion of mucin were restored (Figure 4R) in HFD+ABX+LPJ group compared with those in ABX+HFD group, suggesting that the protective effect of *P. johnsonii* on gut barrier function was not dependent on the presence of the original gut microbes.

### 2.5 The Protective Effects of *P. johnsonii* on Metabolic Disorders and Gut Leak Might be Related to its Metabolites

To investigate whether the metabolites of *P. johnsonii* were contributed to its protection on intestinal barrier integrity, we treated normal colonic epithelial cells (NCM460) with fecal conditioned media (FCM) from ABX treated mice, including Chow+ABX group (CFCM), HFD+ABX group (HFCM) and HFD+ABX+LPJ group (LPJFCM). The gene expressions of *IL6*, *IL1β* and *MCP1 in* LPJFCM treated group were significantly lower than those of HFCM treatment group (Figure S7A–C, Supporting Information). Compared with HFCM group, the expressions of *TJP1* and *OCLN* were higher in cells challenged with LPJFCM (Figure S7D,E, Supporting Information). These results indicated that the metabolites of *P. johnsonii* might be responsible for its beneficial effects on inflammation and intestinal barrier.

### 2.6 Catabolism of BCAAs by *P. johnsonii*

**Figure 5:**
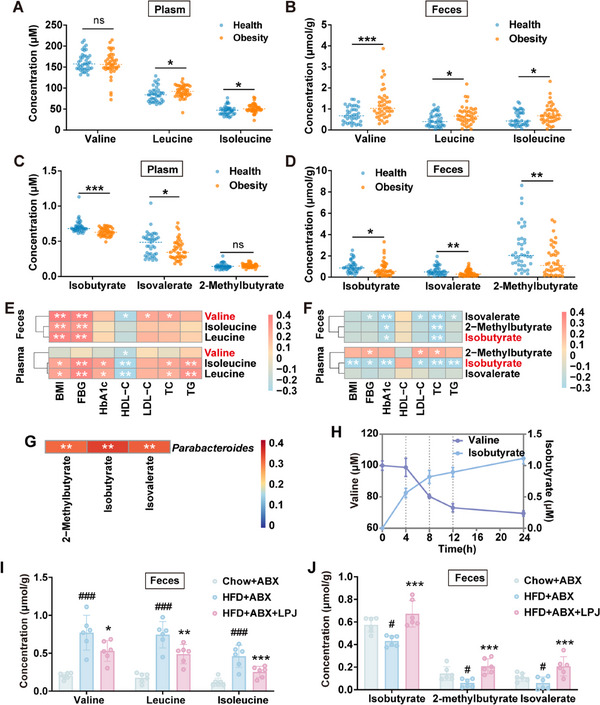
The metabolism of BCAAs to BSCFAs by *P. johnsonii*. A) The levels of BCAAs in the plasma of obese patients. B) The levels of BCAAs in feces of obese patients. C) The levels of BSCFAs in the plasma of obese patients. D) The level of BSCFAs in feces of obese patients. E) Spearman's correlation analysis of BCAAs and blood biochemical indexes in patients. F) Spearman's analysis of correlation between BSCFAs levels and blood biochemical indexes in patients. G) Spearman's analysis of correlation between the abundance of *Parabacteroides* and BSCFAs levels in feces of patients. H) The metabolism of BCAAs by *P. johnsonii* in vitro. I) The levels of BCAAs in the feces of pseudo‐germ‐free mice. J) The levels of BSCFAs in the feces of pseudo‐germ‐free mice. Health, healthy subjects (n = 39); Obesity, obese patients (n = 42); Chow, chow diet‐fed mice; HFD, high fat diet‐fed mice; LPJ, live *P. johnsonii*; ABX, antibiotics; (n = 6). Data are presented as the means ± SD, (A‐D) analyzed by Mann‐Whitney test, (I, J) analyzed by one‐way ANOVA. Chow+ABX versus HFD+ABX, ^#^ *p* < 0.05, ^##^ *p* < 0.01, ^###^ *p* < 0.001; HFD+ABX+LPJ versus HFD+ABX, ^*^ *p* < 0.05, ^**^ *p* < 0.01, ^***^ *p* < 0.001; ns, no significant difference.

BCAAs have been confirmed as potential biomarkers for diseases such as diabetes and cardiovascular disease and they could be catabolized to BSCFAs in the gut (Figure S8A, Supporting Information).^[^ ^3^, ^18^ ^]^ Consistent with previous studies,^[^ ^19^, ^20^ ^]^ we found that the levels of BCAAs in the plasma and feces of obese patients were significantly increased (**Figure** **5**A,B). In contrast, the levels of isobutyrate and isovalerate in the plasma of obese patients were decreased (Figure 5C), and all three types of BSCFAs in the feces were significantly reduced (Figure 5D). Particularly, the level of valine in feces was positively correlated with BMI, FBG, LDL‐C, TC and also negatively correlated with HDL‐C (Figure 5E), whereas the levels of its degradation products isobutyrate was negatively correlated with the level of FBG and TC (Figure 5F), highlighting the abnormal of valine to isobutyrate conversion might be linked with obesity. Of note, the abundance of *Parabacteroides* was significantly positively correlated with the levels of BSCFAs in feces but not those in the plasma (Figure 5G; Figure S8B, Supporting Information). These results suggested that *Parabacteroides* might be responsible for the conversion of BCAAs to BSCFAs in the feces.

To determine the ability of *P. johnsonii* to metabolize BCAAs, whole‐genome analysis was conducted to identify known or hypothesized metabolic gene clusters by gutSMASH. It has been reported that the *porA* gene in *C. sporogenes* catalyzes the conversion of BCAAs into BSCFAs.^[^ ^10^ ^]^ We identified a homologous gene in *P. johnsonii* genome with a 60% similarity to the *porA* gene of the *C. sporogenes* (Figure S8C,D, Supporting Information). Furthermore, after incubation BCAAs with *P. johnsonii*, the contents of BCAAs and BSCFAs in supernatant were measured by LC‐MS. The results showed that the levels of valine (Figure 5H), leucine (Figure S8E, Supporting Information) and isoleucine (Figure S8F, Supporting Information) declined continuously whereas the levels of BSCFAs sustainably increased. Consistent with these in vitro results, LPJ treatment significantly down‐regulated the levels of valine, leucine, isoleucine and up‐regulated the levels of isobutyrate, isovalerate and 2‐methybutyrate in feces of HFD+ABX+LPJ group (Figure 5I,J), demonstrating *P. johnsonii* could catabolize BCAAs into BSCFAs. Similar phenomena could be observed in serum (Figure S8G,H, Supporting Information). In contrast, *P. johnsonii* had little effects on the levels of SCFAs (acetate, propionate, butyrate and caproate) and amino acids (L‐alanine, serine, methionine, phyenyalanine, etc.) (Figure S9, Supporting Information).

### 2.7 Isobutyrate Mirrored the Favorable Effects of *P. johnsonii* on HFD‐fed Mice

**Figure 6:**
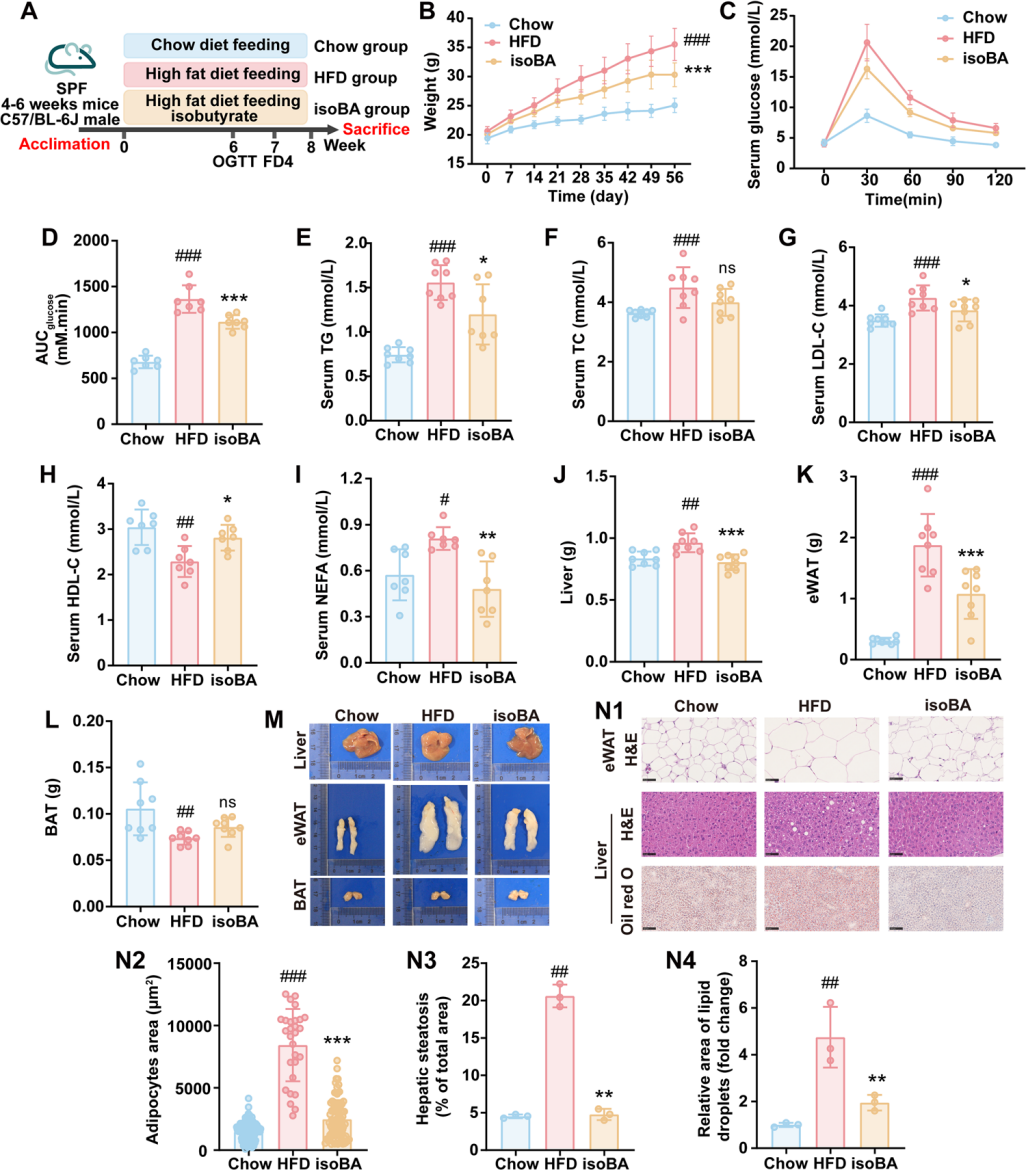
Isobutyrate improved glucose and lipid metabolism disorders in HFD‐fed mice. A) Scheme of the experimental design. B) Body weight change curve. C) Oral glucose tolerance test. D) Area under the curve of OGTT. E‐I) The concentration of TG (E), TC (F), LDL‐C (G), HDL‐C (H) and non‐esterified fatty acids (I) in serum. J‐L) The weight of liver (J), eWAT (K) and BAT (L). M) Representative images of tissue. N) Representative images of H&E and Oil red O staining in liver and WAT (3 mice per group). Scale bars, 50 µm in H&E staining, 100 µm in Oil red O staining. Chow, chow diet‐fed mice; HFD, high fat diet ‐fed mice; isoBA, sodium isobutyrate treated mice (n = 7–8). Data are presented as the means ± SD, analyzed by one‐way ANOVA. Chow versus HFD, ^#^ *p* < 0.05, ^##^ *p* < 0.01, ^###^ *p* < 0.001; isoBA versus HFD, ^*^ *p* < 0.05, ^**^ *p* < 0.01, ^***^ *p* < 0.001, ns, no significant difference.

**Figure 7:**
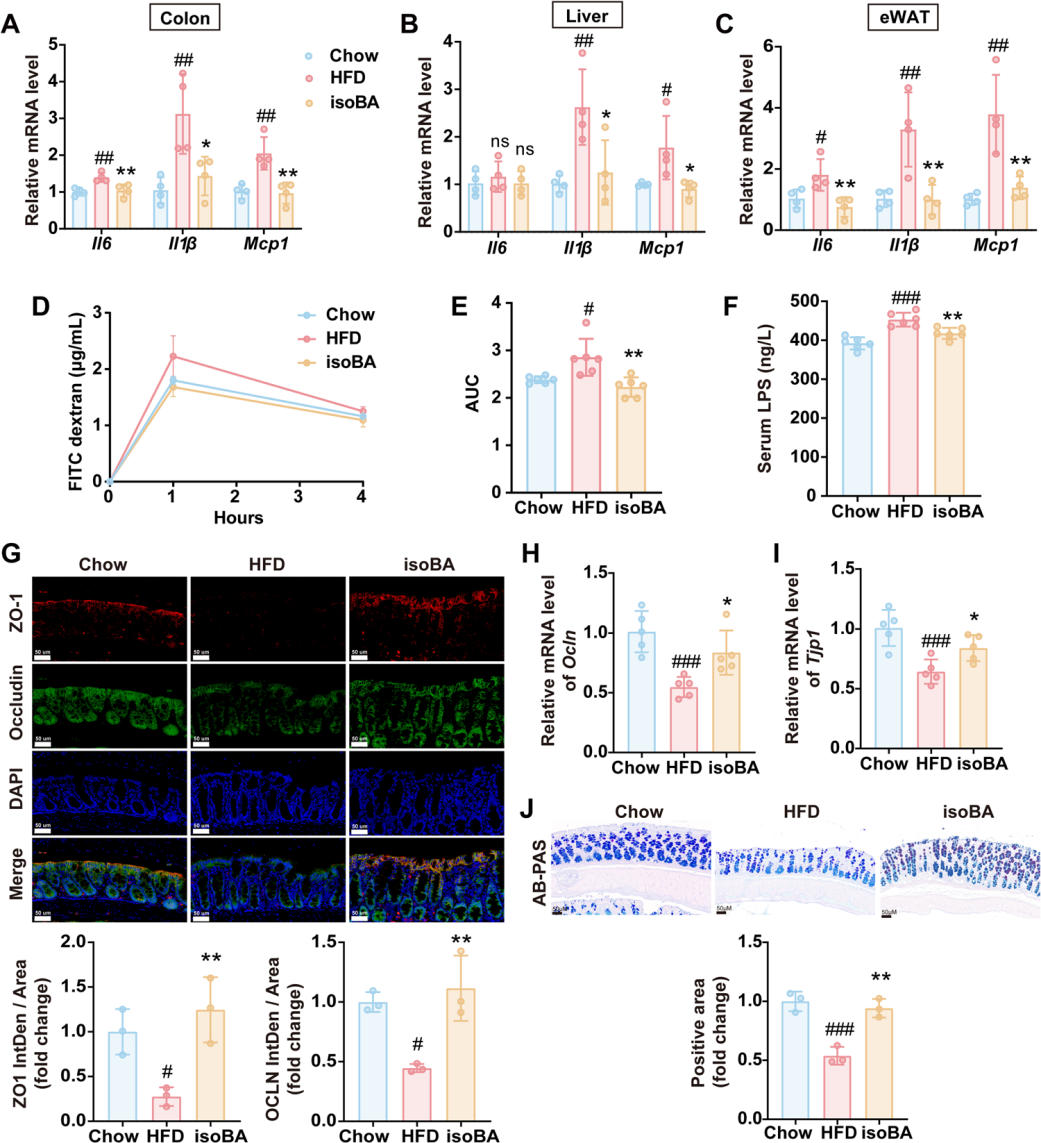
Oral administration of isobutyrate improved inflammation and intestinal mucosal barrier function in HFD‐fed mice. A‐C) Relative mRNA levels of inflammatory factors in colon (A), liver (B) and eWAT (C). D) Intestinal FITC‐dextran test. E) Area under the curve of FITC‐dextran. F) The concentration of LPS in serum. G) Intestinal immunofluorescence of ZO‐1 and Occludin (3 mice per group). Scale bars, 50 µm. H) Relative mRNA level of *Ocln* in colon. I) Relative mRNA level of *Tjp1* in colon. J) AB‐PAS staining of colon (3 mice per group). Scale bars, 50 µm. Chow, chow diet‐fed mice; HFD, high fat diet‐fed mice; isoBA, sodium isobutyrate treated mice (n = 4–6). Data are presented as the means ± SD, analyzed by one‐way ANOVA. Chow versus HFD, ^#^ *p* < 0.05, ^##^ *p* < 0.01, ^###^ *p* < 0.001, ns, no significant difference; isoBA versus HFD, ^*^ *p* < 0.05, ^**^ *p* < 0.01, ^***^ *p* < 0.001, ns, no significant difference.

As isobutyrate in plasma or in feces was negatively correlated with blood glucose and lipid level of obese patient (Figure 5F) and P*. johnsonii* could degrade valine to isobutyrate (Figure 5H), we investigated the effects isobutyrate on HFD‐fed mice (**Figure** **6**A). After 8 weeks of intervention, isobutyrate effectively blocked the increase of body weight (Figure 6B) and the impair of oral glucose tolerance induced by HFD (Figure 6C,D). The blood lipid levels (TG, TC, LDL‐C, HDL‐C and non‐esterified fatty acids) in HFD mice were also restored by isobutyrate (Figure 6E–I). It also reduced the weights of liver and eWAT (Figure 6J–M). In accordance with these results, H&E and Oil Red O staining showed that isobutyrate treatment alleviated lipid accumulation in the liver and reduced the size of adipocytes (Figure 6N). Isobutyrate could decrease the levels of gene expressions related to hepatic lipid *de novo* lipogenesis and uptake whereas promote lipid *β*‐oxidation gene expressions (Figure S10, Supporting Information). In addition, it reduced the mRNA expressions of inflammatory factors (*Il6*, *Il1β* and *Mcp1*) in the colon, liver, and eWAT (**Figure** **7**A–C). These phenomena were accompanied by the improvement of gut epithelial barrier integrity as indicated by the reduced FITC‐dextran (Figure 7D,E) and LPS (Figure 7F) levels in serum, the upregulation of Occludin and ZO‐1 (Figure 7G–I), and the increased goblet cells (Figure 7J) after isobutyrate treatment. These data collectively indicated that isobutyrate mirrored the favorable effects of *P. johnsonii* on HFD‐fed mice.

### 2.8 Isobutyrte Promoted the Expression of FGF1 in Intestinal Epithelial Cells

**Figure 8:**
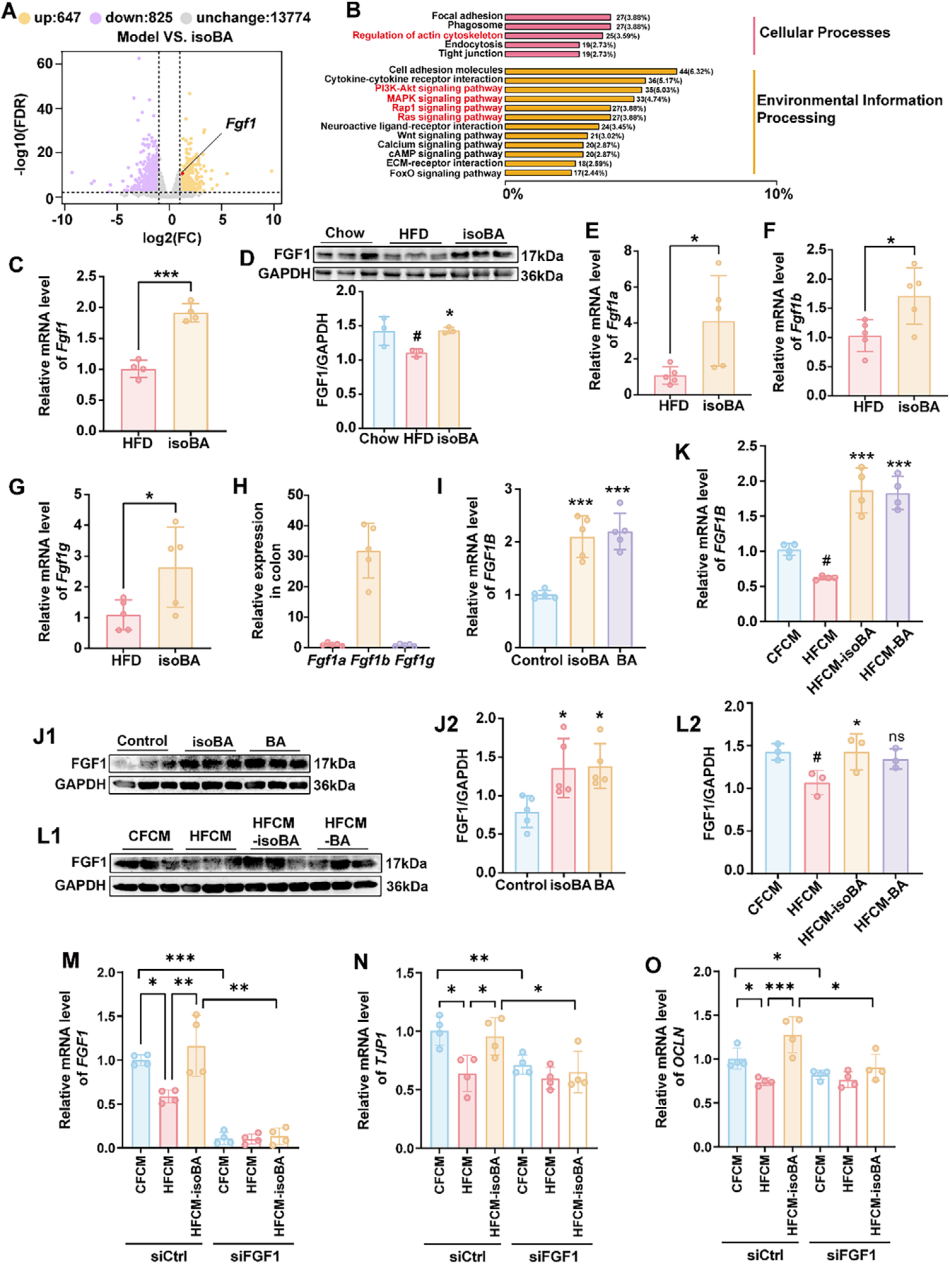
Isobutyrate promoted the expression of FGF1 in colon of HFD‐fed mice or in NCM460 cells. A) Volcano plot of differentially expressed genes in the colon after isobutyrate treatment, n = 5. B) KEGG analysis of differentially expressed genes. C) The effects of isobutyrate on mRNA expression of *Fgf1* in the colon of HFD‐fed mice, n = 5. D) The effects of isobutyrate on FGF1 expression in the colon of HFD‐fed mice, n = 3. E‐G) The effects of isobutyrate on mRNA expressions of *Fgf1a* (E), *Fgf1b* (F) and *Fgf1g* (G) in the colon of HFD‐fed mice, n = 5. H) The relative abundance of *Fgf1a*, *Fgf1b* and *Fgf1g* in the colon of mice. I,K) The effects of isobutyrate and butyrate on the expression of *FGF1B* splicing variant in NCM460 cells, n = 4 or 5. J,L) The effects of isobutyrate and butyrate on the expression of FGF1 in NCM460 cells, n = 3 or 5. M‐O) Relative mRNA level of *FGF1*, *TJP1* and *OCLN* after *FGF1* knock down in NCM460 cells. HFCM: Fecal conditioned media of HFD mice; CFCM: Fecal conditioned media from Chow group; HFCM‐isoBA: Fecal conditioned media from HFD mice treated with isoBA. Data are presented as the means ± SD, analyzed by one‐way ANOVA for 3 or more groups, analyzed by unpaired t test in (C, E–G). Chow versus HFD, CFCM versus HFCM, ^#^ *p* < 0.05, ^##^ *p* < 0.01, ^###^ *p* < 0.001; isoBA versus HFD, HFCM‐isoBA versus HFCM, HFCM‐BA versus HFCM, isoBA versus Control, BA versus Control, ^*^ *p* < 0.05, ^**^ *p* < 0.01, ^***^ *p* < 0.001, ns, no significant difference.

As LPJ treatment remarkably increased the level of isobutyrate in feces but not in serum of HFD‐fed mice under ABX intervention (Figure 5J; Figure S8H, Supporting Information), we speculated that *P. johnsonii* and isobutyrate exerted their favorable effects on metabolic disorder mainly through the maintenance of gut epithelial barrier integrity. Thus, to gain molecular insights into the protective effects of isobutyrate and *P. johnsonii* in colon, we conducted RNA sequencing of colon tissues from HFD‐fed mice with or without isobutyrate. We observed 647 upregulated and 825 downregulated genes in isobutyrate‐treated mice compared to model group (q <0.01, Fold change > 2, **Figure** **8**A). Among them, we found that the expression of the *Fgf1* gene was significantly increased after isobutyrate treatment. KEGG pathway analysis was performed on the differentially expressed genes (Figure 8B). FGF1 belonged to the growth factor and it has been reported that FGF1 could promote the repair of intestinal epithelial cells and block TLR4‐mediated intestinal epithelial injury.^[^ ^21^ ^]^ To verify the significance of FGF1 in intestinal inflammation and barrier function, we evaluated its expression in ulcerative colitis (UC) or crohn's disease (CD) by four independent gene expression comprehensive datasets. Expression of *FGF1* was significantly downregulated in UC and CD (Figure S11A–C, Supporting Information), and there was also significant difference between the non‐inflammatory and inflammatory states in UC (Figure S11D, Supporting Information). Furthermore, the results of QPCR and Western blotting showed that isobutyrate treatment increased the mRNA and protein expressions of FGF1 in the colon compared with those in HFD group (Figure 8C,D). Consistent results were also observed in ABX‐treated mice gavaged with LPJ (Figure S12A, Supporting Information).

It has been reported that there are three splicing variants in mice, *Fgf1a*, *Fgf1b*, and *Fgf1g*.^[^ ^22^ ^]^ We found that isobutyrate could promote the expressions of these splicing variants in intestine (Figure 8E–G). Among these, the abundance of *Fgf1b* was the highest (Figure 8H). Meanwhile, we also investigated whether isobutyrate could regulate *FGF1* in normal human colonic epithelial cells NCM460. Unlike in mice, human‐derived *FGF1* has four splicing variants, namely *FGF1A*, *FGF1B*, *FGF1C*, and *FGF1D*. In NCM460 cells, we detected three splicing variants, *FGF1A*, *FGF1B*, and *FGF1D*. The results showed that isobutyrate selectively promoted the mRNA expression of the *FGF1B* (Figure 8I; Figure S12B,C, Supporting Information) and increased the FGF1 protein level (Figure 8J). Furthermore, after HFCM treatment, the expression of *FGF1B* and protein level of FGF1 in NCM460 cells were inhibited and these trends were restored by isobutyrate or butyrate supplement (Figure 8K,L).

To verify the role of *FGF1* in intestinal barrier function, we knocked down the *FGF1* gene in NCM460 cells (the efficiency was ≈90% at the mRNA levels, as shown in Figure 8M). The fecal conditioned media of mice on a high‐fat diet (HFCM) inhibited the transcription of *FGF1* (Figure 8M), *TJP1* (Figure 8N), and *OCLN* (Figure 8O), and isobutyrate promoted their expression. However, compared with the negative control group, the promoting effect of isobutyrate on *TJP1* and *OCLN* was significantly blocked after *FGF1* knockdown (Figure 8N,O). These results suggested FGF1 played an important role in the protective effects of isobutyrate and *P. johnsonii* on gut epithelial barrier.

### 2.9 Isobutyrate Inhibited HDAC3 Activity and Promoted H3K14 Acetylation in the Promoter Region of FGF1B

**Figure 9:**
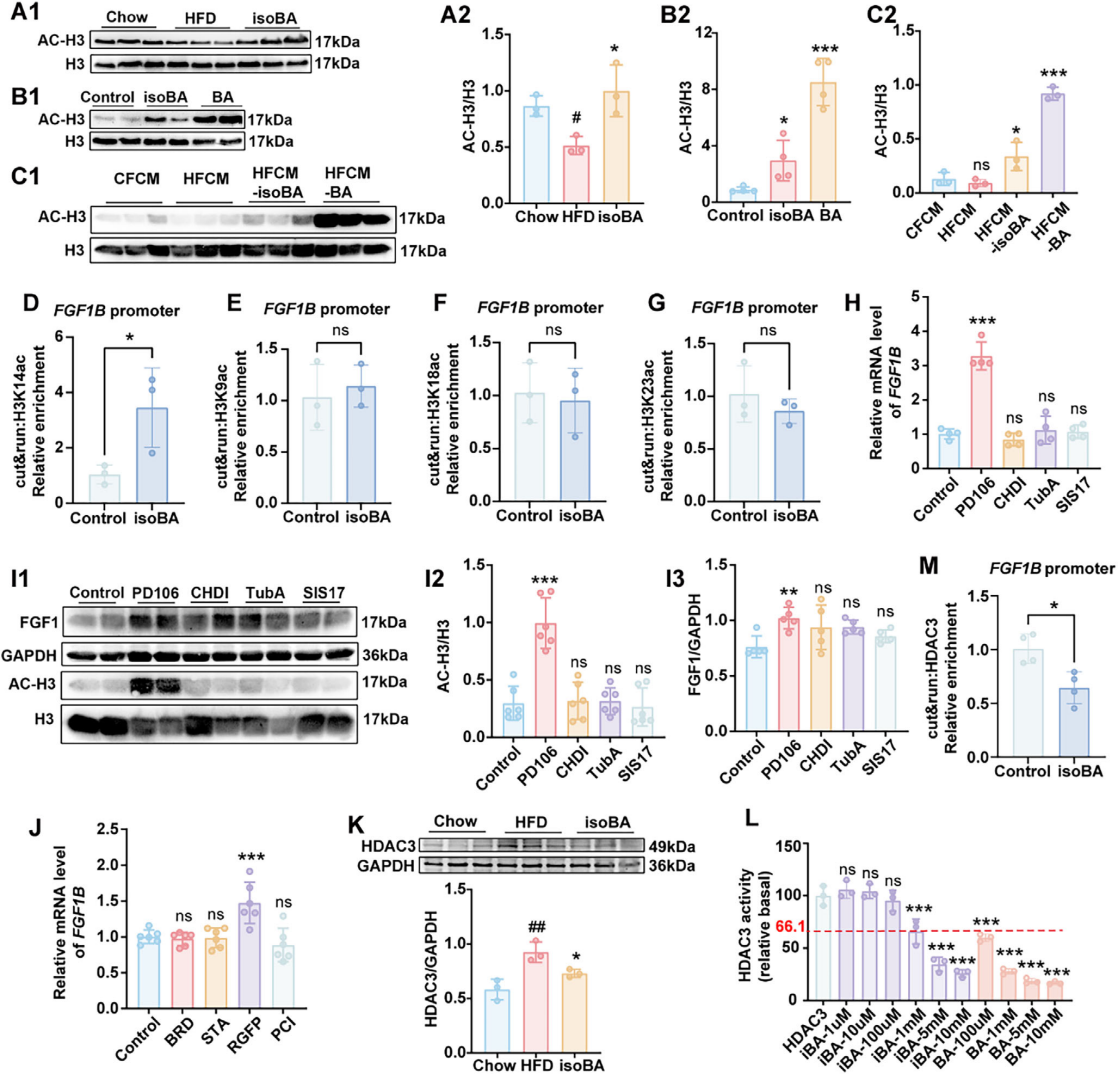
The effects of isobutyrate on HDAC3 activity and H3 acetylation. A) The level of H3 acetylation in the colon of mice, n = 3. B,C) The effect of isobutyrate on H3 acetylation in NCM460 cells, n = 3 or 5. D‐G) Cut & run assay showing H3K14ac (D), H3K9ac (E), H3K18ac (F), H3K23ac (G) occupancy on the *FGF1B* promoter in NCM460 cells with or without isobutyrate. H) Effects of HDACs inhibitors on *FGF1B* in NCM460 cells, n = 4. I) Effects of HDACs inhibitor on FGF1 and H3 acetylation in NCM460 cells, n = 5 or 6. J) Effects of HDAC I inhibitors on *FGF1B* in NCM460 cells, n = 6. K) The expression of HDAC3 in the colon of mice (n = 3). L) Enzyme activity analysis of HDAC3, n = 3. M) Cut & run assay showing HDAC3 occupancy on the *FGF1B* promoter in NCM460 cells with or without isobutyrate. Data are presented as the means ± SD, analyzed by one‐way ANOVA for 3 or more groups or by unpaired t test for 2 groups. Chow versus HFD, ^#^ *p* < 0.05, ^##^ *p* < 0.01; isoBA versus HFD, Control versus other groups, ^*^ *p* < 0.05, ^**^ *p* < 0.01, ^***^ *p* < 0.001; CFCM versus HFCM, ns, no significant difference; HFCM versus HFCM‐isoBA or HFCM‐BA, ^*^ *p* < 0.05, ^***^ *p* < 0.001.

SCFAs have been shown to strengthen the integrity of the epithelia barrier by acting on G protein‐coupled receptor (GPR) 43 and GPR109A or histone deacetylases (HDACs).^[^ ^23^ ^]^ As isobutyrate is an isomer of butyrate, we wondered to know whether GRP43 and GPR109A or HDACs contributed to the regulatory effect of isobutyrate on *FGF1B*. First, we found isobutyrate enhanced the expression of *FGF1B* in NCM 460 cells and this trend was not altered by GLPG0974 (GPR43 antagonist) or Mepenzolate bromide (GPR109A antagonist) co‐treatment (Figure S12D, Supporting Information), suggesting that the effect of isobutyrate on *FGF1B* was not dependent on GPR43 or GPR109A. Second, we examined the acetylation level of H3 in the intestinal tissues of mice. The results of Western blotting showed that HFD inhibited the expression of acetylated H3, while treatment with isobutyrate promoted the acetylation of H3 (**Figure** **9**A1,A2). In NCM460 cells, isobutyrate incubation could also enhance H3 acetylation (Figure 9B,C). Cut & run assay confirmed isobutyrate induced the enrichment of acetylated H3K14 at the *FGF1B* promoter (Figure 9D), rather than that of H3K9, H3K18, or H3K23 (Figure 9E–G). These results indicated the up‐regulation of *FGF1B* transcription by isobutyrate might be related to increase of H3K14 histone acetylation.

To investigate which HDACs affects the acetylation of *FGF1B* promoter, HDAC I inhibitor PD106, HDAC IIa inhibitor CHDI‐390576, HDAC IIb inhibitor Tubastatin A, and HDAC IV inhibitor SIS17 were used to treat NCM460 cells. The results showed that only PD106 promoted the transcription of the *FGF1B* (Figure 9H) and the expressions of acetylated H3 and FGF1 (Figure 9I1‐I3), highlighting the role of Class I HDACs in FGF1 expression. Considering Class I HDACs includes HDAC1, HDAC2, HDAC3, and HDAC8, the specific inhibitors of HDAC1 and HDAC2 (BRD – 6929), HDAC2 (STA), HDAC3 (RGFP966), and HDAC8 (PCI – 34051) were further incubated with NCM460 respectively. We found that only the HDAC3 inhibitor RGFP966 could promote the expression of *FGF1B* (Figure 9J). Meanwhile, western blot results showed HFD promoted the expression of HDAC3 in the intestine of mice, whereas isobutyrate consumption inhibited its expressions (Figure 9K). Consistent results were also observed in ABX‐treated mice gavaged with LPJ (Figure S12E, Supporting Information). In line with these results, isobutyrate treatment at 1 and 10 mM inhibited the HDAC3 enzyme activity by 33.9% and 74.1% (Figure 9L), respectively. Cut & run assay further revealed isobutyrate could decrease the level of HDAC3 at the *FGF1B* promoter (Figure 9M). Taken together, these data revealed that isobutyrate inhibited enzymatic activity of HDAC3 to enhance *FGF1B* transcription.

### 2.10 Stachyose Promoted the Growth of *P. johnsonii* and Improved Metabolic Disorder

**Figure 10:**
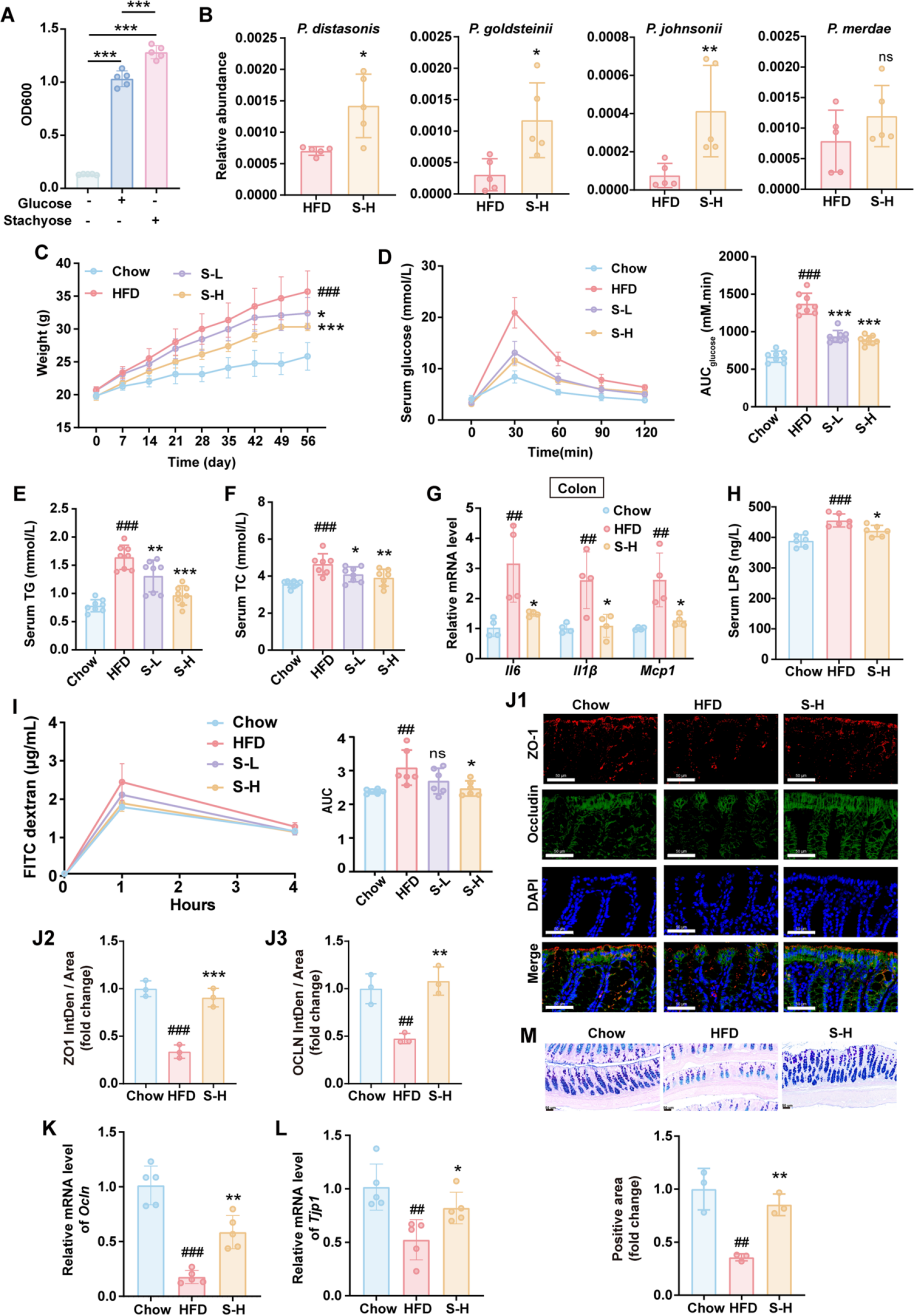
The effects of stachyose on *parabacteroides* and metabolic disorder in HFD‐fed mice. A) The effect of stachyose on the growth of *P. johnsonii* in vitro (n = 5). B) The regulation of stachyose on the abundance of *parabacteroides* in HFD‐fed mice. C) Body weight change curve. D) Oral glucose tolerance test. E) The concentration of TG in serum. F) The concentration of TC in serum. G) Relative mRNA levels of inflammatory factors in colon. H) The concentration of LPS in serum. I) Intestinal FITC‐dextran test and area under the curve of FITC‐dextran. J) Intestinal immunofluorescence of ZO‐1 and Occludin (3 mice per group). Scale bars, 50 µm. K) Relative mRNA level of *Ocln* in colon. L) Relative mRNA level of *Tjp1* in colon. M) AB‐PAS staining of colon (3 mice per group). Scale bars, 50 µm. Chow, chow diet‐fed mice; HFD, high fat diet‐fed mice; S‐L, low dose of stachyose treated HFD‐fed mice; S‐H, high dose of stachyose treated HFD‐fed mice, n = 5 – 8. Data are presented as the means ± SD, analyzed by Mann‐Whitney test in (B) and by one‐way ANOVA for 3 or more groups. Chow versus HFD, ^#^ *p* < 0.05, ^##^ *p* < 0.01, ^###^ *p* < 0.001; S‐L or S‐H versus HFD, ^*^ *p* < 0.05, ^**^ *p* < 0.01, ^***^ *p* < 0.001; ns, no significant difference.

The ability of polysaccharides and oligosaccharides to maintain homeostasis of gut microbe has been demonstrated.^[^ ^24^, ^25^ ^]^ We have revealed that *P. johnsonii* could ameliorate inflammation and regulating lipid metabolism in HFD‐fed mice. Based on these findings, we wanted to find potential candidate which could promote the growth of *P. johnsonii* and could be used as prebiotics. It has been reported stachyose intervention remarkably increased the relative abundance of *Parabacteroides* in human and KKAy mice.^[^ ^26^, ^27^ ^]^ Therefore, we investigated the ability of stachyose on the growth of *P. johnsonii*. The results showed that stachyose significantly promoted the growth of *P. johnsonii* in vitro (**Figure** **10**A). In HFD‐fed mice, the relative abundances of *P. distasonis*, *P. goldsteinii*, and *P. johnsonii* were increased by 1.9 times, 3.7 times, and 7.2 times respectively after stachyose treated, while it had no significant impact on *P. merdae* (Figure 10B). Oral gavage with stachyose reduced the weight gain induced by HFD (Figure 10C) and improved metabolism (Figure 10D–F; Figure S13A–C, Supporting Information). Stachyose reduced the mRNA expressions of inflammatory factors in colon (Figure 10G) and also alleviated inflammation in liver and eWAT at different degrees (Figure S13D,E, Supporting Information). Gut epithelial barrier function which was impaired by HFD was also ameliorated after stachyose intervention (Figure 10H–M). Therefore, as a potential prebiotic, stachyose could promote the growth of *P. johnsonii* and alleviate metabolic disorder and improve the intestinal barrier integrity.

### 2.11 Stachyose Enhanced the Transformation of BCAAs to BSCFAs and Activated *Fgf1* Transcription

**Figure 11:**
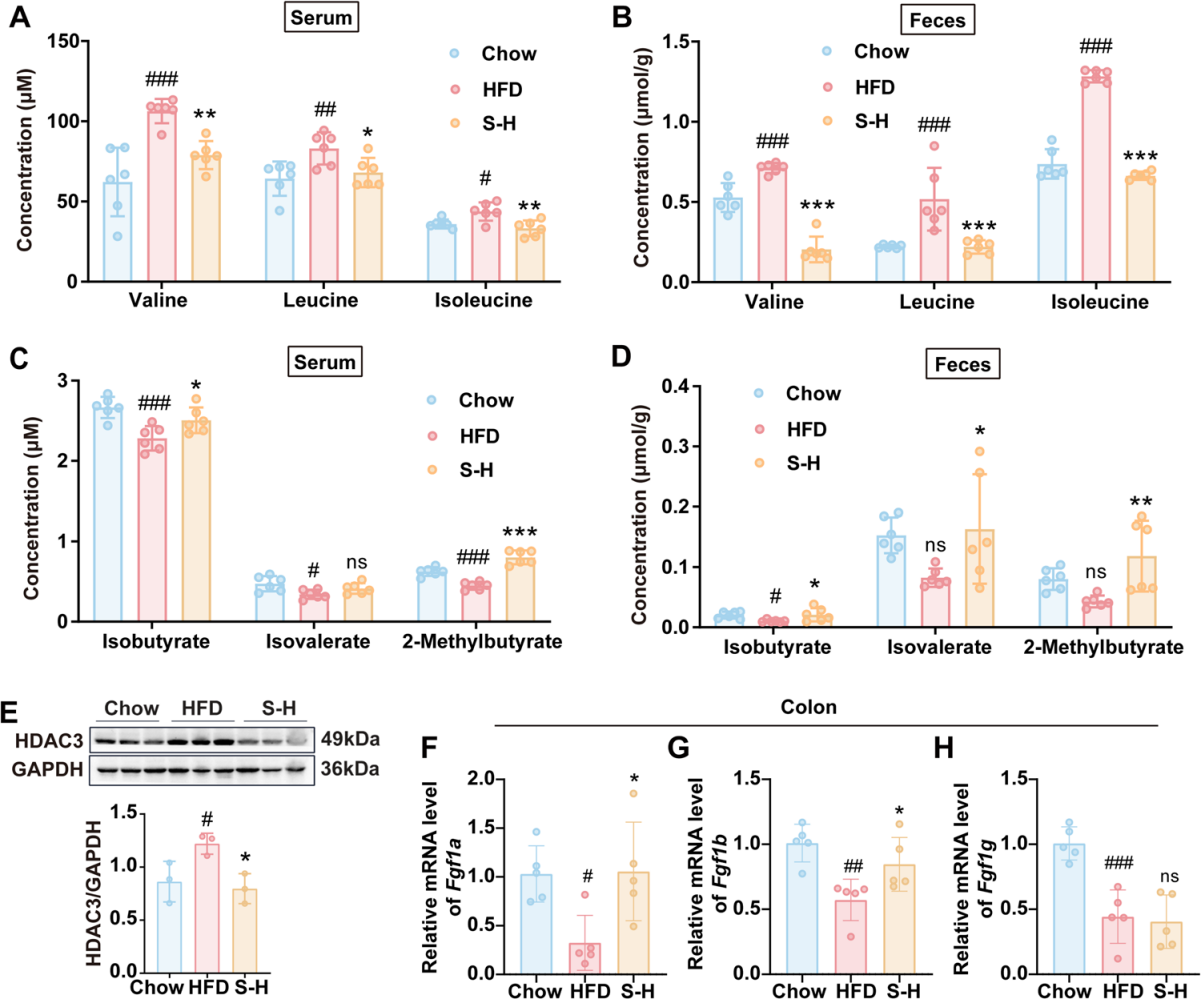
The effects of stachyose on the metabolism of BCAAs and the expressions of *Fgf1* and HDAC3 in HFD‐fed mice. A) The concentrations of BCAAs in serum. B) The concentrations of BCAAs in feces. C) The concentrations of BSCFAs in serum. D) The concentrations of BSCFAs in feces. E) The expression of HDAC3 in the colon of mice (n = 3). F‐H) Effect of stachyose on the expression of *Fgf1* in colon. Chow, chow diet‐fed mice; HFD, high fat diet‐fed mice; S‐H, high dose of stachyose treated HFD‐fed mice, n = 5 – 6. Data are presented as the means ± SD, analyzed by one‐way ANOVA for 3 groups. Chow versus HFD, ^#^ *p* < 0.05, ^##^ *p* < 0.01, ^###^ *p* < 0.001; S‐H versus HFD, ^*^ *p* < 0.05, ^**^ *p* < 0.01, ^***^ *p* < 0.001; ns, no significant difference.

As stachyose could regulate *P. johnsonii*, we wondered to know its effects on BCAAs catabolism. LC‐MS results showed stachyose significantly reduced the levels of BCAAs in serum and feces (**Figure** **11**A,B) and simultaneously increased the levels of BSCFAs (Figure 11C,D). Meanwhile, stachyose treatment inhibited the expression of HDAC3 in the colon of HFD‐fed mice (Figure 11E). We also detected mRNA expressions of *Fgf1* in the intestine. As shown in Figure 11F–H, the levels of *Fgf1a*, *Fgf1b*, and *Fgf1g* in colon were decreased in HFD group, whereas the expressions of *Fgf1a* and *Fgf1b* were reversed by stachyose. A recent study has shown that stachyose can directly bind to HSP90b in intestinal epithelial cells to regulate the microbiome.^[^ ^26^ ^]^ To confirm whether stachyose regulates *FGF1* through gut microbiota metabolites or directly acting on intestinal epithelial cells, we incubated stachyose with NCM460 cells in vitro and detected the expression of *FGF1B*. The results showed that stachyose at concentrations ranging from 0.1 mg mL^−1^ to 3 mg mL^−1^ had no effect on the expression of *FGF1B* in NCM460 cells (Figure S14, Supporting Information). Taken together, these data suggested that the health benefits of stachyose might be related to its boosting effects on the growth of *P. johnsonii* and BCAAs to BSCFAs conversion, thereby inhibiting HDAC3 and activating the expression of *Fgf1* in the intestine.

## 3 Discussion

With the rapid advancement of microbiome research, *Parabacteroides*. Spp., with an average abundance of 1.27% in human flora, has emerged as a significant focus in the study of obesity.^[^ ^28^ ^]^ Notably, species like *P. distasonis* and *P. goldsteinii* have garnered particular attention. It has been reported *P. distasonis* and *P. goldsteinii* are potential probiotics for patients with obesity, inflammatory bowel disease, and metabolic syndrome.^[^ ^15^, ^29^, ^30^ ^]^ A recent study showed *P. merdae* could protect against cardiovascular damage.^[^ ^11^ ^]^ However, there are few reports on the function of *P. johnsonii*. Our results revealed the abundance of *P. johnsonii* significantly correlated with blood lipid levels in obese patients, suggesting that changes of *P. johnsonii* might be involved in the progression of obesity. This finding expanded the understanding of *Parabacteroides* spp. However, we did not have enough data to analyze correlation between *P. johnsonii* and liver steatosis at present. Examining the relationship between the severity of hepatic steatosis in obese patients and *P. johnsonii* will better elucidate the beneficial effects of *P. johnsonii* in future. Meanwhile, *P. johnsonii* could regulate hepatic lipid uptake and *β*‐oxidation in HFD‐fed mice, at least partly accounting for its metabolic benefits. In addition, given that systemic lipid homeostasis is a highly integrated process involving multiple organs (liver, adipose tissue, skeletal muscle, intestine),^[^ ^31^, ^32^ ^]^ its impact on lipid metabolism in other organs, such as skeletal muscle and adipose tissue, remains to be investigated. Directly measuring fatty acid flux, tissue‐specific fatty acid oxidation, and VLDL kinetics will provide stronger mechanistic support for the beneficial effects of *P. johnsonii*, and we highlight these as important directions for future research. Furthermore, our data also showed a significant inverse relationship (*p< 0.01*) between FBG and fecal *P. johnsonii* abundance in obese patients (Figure 1G), indicating *P. johnsonii* might have antidiabetic properties. *P. johnsonii* could alleviate insulin resistance in HFD‐fed mice, evidenced by the OGTT test. We did not observe significant change on FBG in HFD‐fed mice. This might be due to the duration of HFD only lasting six weeks. In future studies, we could extend the duration of HFD feeding to examine the effect of *P. johnsonii* on FBG improvement. Concurrently, diabetic patients may be enrolled to investigate the therapeutic effects of *P. johnsonii* on diabetes.

Metabolites produced by the gut microbiota are crucial in regulating the complex interaction between the gut microbiota and the host.^[^ ^33^ ^]^ In order to elucidate how *P. johnsonii* exerted health benefit, we investigated its metabolites. Considering the strong link between BCAAs catabolism and obesity, we paid special attention to the changes of BCAAs in HFD‐fed mice after oral administrated with *P. johnsonii*. The levels of BCAAs in the feces of HFD‐fed mice were increased along with the decrease of BSCFAs levels whereas *P. johnsonii* reversed these trends. Together with the in vitro results, we revealed *P. johnsonii* had the capacity for enhancing the BCAAs to BSCFAs conversion. Although it has been proved *porA* gene in *C. sporogenes* catalyzed the conversion of BCAAs into BSCFAs and *P. johnsonii* genome with a 60% similarity to the *porA* gene of the *C. sporogenes*, the key microbial enzymes in *P. johnsoni* responsible for the conversion still requires clarification. In addition, it has been reported *P. distasonis* alleviated obesity and metabolic dysfunctions via production of succinate and secondary bile acids.^[^ ^14^ ^]^ Whether *P. johnsonii* has the same effects as *P. distasonis* on succinate and bile acids still needs to be investigated in future. In our study, we found *P. johnsonii* could regulate glucose tolerance and reduce blood lipid levels (Figure S4, Supporting Information) in normal chow diet‐fed mice where gut integrity is not altered. In addition to affecting intestinal barrier integrity, *P. johnsonii* may also exert direct effects on tissues such as the liver and adipose tissue via its metabolites, including short‐chain fatty acids and bile acids.

Unlike BCAAs, there are few studies on the role of BSCFAs in obesity, and the effects of BSCFAs remains controversial.^[^ ^34^, ^35^ ^]^ A study revealed the gluconeogenic potential of isobutyrate and isovalerate in hepatocytes.^[^ ^36^ ^]^ However, another study showed that BSCFAs could improve insulin sensitivity via regulating the lipid and glucose metabolism in adipocytes.^[^ ^37^ ^]^ Therefore, systemic investigations were conducted to explore the function of BSCFAs in this study. We found the levels of BSCFAs in fecal samples were decreased in obese patients along with the elevated BCAAs. Similar results were found in HFD‐fed mice. These trends were reversed by *P. johnsonii* intervention. Among three BSCFAs, isobutyrate had the highest concentration and was correlated to the symptoms of obese patients, underpinning its clinical significance. This interesting finding prompted us to further study the potential mechanism of isobutyrate. Exogenous administration of isobutyrate conferred the favorable effects of *P. johnsonii* on HFD‐fed mice. Since *P. johnsonii* intervention only elevated the level of isobutyrate in feces but not in serum of HFD‐fed mice, we speculated that isobutyrate exerted its beneficial effects in the intestine. Through RNA sequencing of colon tissue from HFD‐fed mice with or without isobutyrate, we found that isobutyrate significantly activated the transcription of *Fgf1* in the colon. FGF1, also known as acidic FGF, is found throughout the body. It's highly expressed in the brain and pituitary gland, and is also present in significant amounts in peripheral tissues such as adipose tissue, the liver, skeletal muscle, heart, lungs, and kidneys.^[^ ^38^ ^]^ FGF1 acts as an autocrine/paracrine hormone and has emerged as a new way to manage type 2 diabetes. Even though FGF1 is expressed in many tissues, only the FGF1 from adipose tissue and the brain are known to play a role in regulating metabolism.^[^ ^39^ ^]^ We found the level FGF1 in colon was upregulated by isobutyrate. As it was reported FGF1 always binds to heparan sulphate proteoglycans which prevents it from entering the circulation,^[^ ^39^ ^]^ we hypothesized FGF1 exerts its effects in colon instead of entering to adipose and brain. Previous research has been proved FGF1 could block TLR4‐mediated intestinal epithelial injury and promote the expression of ZO1.^[^ ^21^ ^]^ We further revealed the expression of *FGF1* was significantly downregulated in UC and CD patients. These results indicated isobutyrate‐mediated FGF1 secretion could be the underlying mechanism for the efficacy of *P. johnsonii*. In addition to *Fgf1*, RNA sequencing showed many genes related to immune signaling were also changed by isobutyrate. Continued researches on these genes are still needed to enhance our understanding of the functions of BSCFAs.

The human *FGF1* gene is over 120 kb long and contains four distinct splicing variants (*FGF1*A, B, C, D) regulating the expression of FGF1 in a tissue‐specific context.^[^ ^40^ ^]^ Meanwhile, there are three splicing variants in mice (*Fgf1a*, *Fgf1b*, and *Fgf1g*).^[^ ^22^ ^]^ We found *Fgf1b* transcript predominated in mice colon and isobutyrate elevated the mRNA of *Fgf1b*. Isobutyrate also selectively increased the mRNA of *FGF1B* in human NCM640 cells. SCFAs function via two primary pathways: inhibiting HDACs and signaling through GPCRs. Given this, we aimed to explore how isobutyrate exerts its effects. Blockade of GPCR signaling did not change the effects of isobutyrate on *Fgf1b* whereas HDAC3 inhibitor recapitulated the role of isobutyrate, suggesting the effect of isobutyrate on *Fgf1b* was mediated in a HDAC3 dependent manner but not through GPCR signaling. These results were further supported by HDAC3 enzyme activity and cut & run assay, which showed isobutyrate significantly inhibited HDAC3 activity and promoted H3K14 acetylation at the *FGF1B* promoter. This finding was in accordance with previous reports that HDAC3 inhibition led to the H3K14 acetylation at the *Fgf1b* promoter in the brain of mice.^[^ ^41^ ^]^ Consistently, *P. johnsonii* intervention also suppressed HDAC3 to active *Fgf1b* transcription, indicating *P. johnsonii* could attenuate metabolic dysfunction, at least by enhancing the BCAAs to BSCFAs conversion.

## 4 Conclusion

We provided evidence for the beneficial effects of the gut microbe *P. johnsonii* against metabolic disorders. *P. johnsonii* enhanced the conversion of BCAAs to BSCFAs, especially the catabolism of valine to isobutyrate. Isobutyrate activated *Fgf1* transcription through inhibition of HDAC3 to increase H3K14 acetylation, thereby improving intestinal permeability and systemic inflammation. Additionally, the natural product stachyose showed a favorable ability to prevent the development of obesity by facilitating the growth of *P. johnsonii*. Our study presents the gut microbe *P. johnsonii* as a probiotic and stachyose as a prebiotic agent for the treatment of metabolic disorders.

## 5 Experimental Section

### Recruitment of Obese Patients and Healthy Participants

The human studies were all approved by the Ethics Committee of Nanjing Hospital of Chinese Medicine in accordance with the World Medical Association's Declaration of Helsinki (KY2022424). Written informed consent was obtained from all participants before recruitment. Feces and plasma from 42 obese patients (BMI≥25 kg/m^2^) and 39 age‐ and sex‐matched healthy subjects (BMI 19–24 kg/m^2^) were collected from the Department of Infectious Diseases, Nanjing Hospital of Chinese Medicine Affiliated to Nanjing University of Chinese Medicine (Table S1, Supporting Information). Patients were excluded if they had diabetes, kidney disease, hypertension, cardiovascular diseases, intestinal diseases, liver diseases, lung diseases, or had used antibiotics, antiviral drugs, and probiotics in the past three months. After fasted, the blood and fecal samples were collected and stored at ‐80 °C for subsequent analysis.

### Animal Experimental Design

All animal experiments were conducted in accordance with the Provisions and General Recommendation of Chinese Experimental Animals Administration Legislation and this study was approved by Animal Ethics Committee of China Pharmaceutical University (No. 2022‐11‐014). Male C57BL/6J mice at six – week – old (Charles River Laboratories, Beijing, China) were used in the following experiments. The mice were maintained with a standard laboratory diet and free access to tap water in a controlled environment with room temperature (22 °C ± 1 °C), humidity (65% ± 5%) and a 12:12 h light/dark cycle. The chow diet (Research Diet, D12450B, 10% calories from fat) or high fat diet (HFD, Research Diet, D12492, 60% calories from fat) or maintenance diet (Jiangsu‐Xietong, Inc., Nanjing, China) were used in these experiments.

When evaluating the effectiveness of *P. johnsonii* in mice fed HFD, the animals were randomly divided into four groups and administrated for 6 weeks: (1) Chow group (n = 6), (2) HFD group (n = 6), (3) LPJ group (n = 6), gavage with live *P. johnsonii*, 2 × 10^9 CFUs mL^−1^, 0.2 ml per day per mouse), (4) KPJ group (n = 6, gavage with heat‐killed *P. johnsonii*, 2 × 10^9 CFUs mL^−1^, 0.2 ml, per day per mouse). The mice in chow group were given a chow diet and mice in other groups were fed with HFD during the experimental period.

For evaluating the effectiveness of *P. johnsonii* on HFD‐fed pseudo‐germ‐free mice, mice were treated with antibiotics cocktail [ABX; including vancomycin (1 mg mL^−1^), neomycin sulfate (5 mg mL^−1^), metronidazole (5 mg mL^−1^), streptomycin (5 mg mL^−1^), and ampicillin (5 mg mL^−1^), 0.2 ml per mouse per day] for a week, and then randomized into three groups: 1) Chow+ABX group (n = 8), 2) HFD+ABX group (n = 8), 3) HFD+ABX+LPJ group (n = 8, gavage with live *P. johnsonii*, 2 × 10^9 CFUs mL^−1^, 0.2 ml per day per mouse). Before feeding with HFD, gavaged with live *P. johnsonii* in HFD+ABX+LPJ group for a week and collecting fecal samples (just prior administration of live *P. johnsonii*). For the next 6 weeks, the mice in Chow+ABX group received a chow diet, those in HFD+ABX group received a high fat diet, those in HFD+ABX+LPJ group received a high fat diet with administration of live *P. johnsonii*.

For evaluating the effectiveness of *P. johnsonii* on normal mice, mice were randomized into two groups for a duration of 6 weeks: (1) Chow group (n = 6), (2) LPJ group (n = 6, gavage with live *P. johnsonii*, 2 × 10^9 CFUs mL^−1^, 0.2 ml per day per mouse). The mice in both groups were given maintenance diet.

For the isobutyrate or stachyose efficiency assays on HFD‐fed mice, mice were randomized into three or four groups for a duration of 8 weeks: (1) Chow group (n = 8), (2) HFD group (n = 8), (3) treatment groups (n = 8). The treatment groups were orally administered with sodium isobutyrate (500 mg kg^−1^, every other day) or low dose of stachyose (100 mg kg^−1^, six times a week) or high dose of stachyose (300 mg kg^−1^, six times a week). The mice in chow group were given a chow diet and mice in other groups were fed with HFD during the experimental period.

### Cell Culture and Treatments

Normal intestinal epithelium cell line NCM460 (ATCC, Rockville, MD, USA) were cultured in DMEM medium containing 20% (v/v) fetal bovine serum. All the experiments were carried out when the cell density reached 80%. After treatment for 12 h, the cells were harvested for further analysis.

For isobutyrate or butyrate treatment assays, cells were treated with DMEM containing 10 mM sodium isobutyrate (Macklin, Shanghai, China) or sodium butyrate (Aladdin, Shanghai, China).

For fecal conditioned media (FCM) treatment assays, FCM preparation was performed as previously reported.^[^ ^42^ ^]^ Feces (30 mg) were suspended in 600 µL cold DMEM media by homogenizer. The suspended media was filtered twice. The cells were treated with medium containing FCM (1:100, FCM: medium) without or with 10 mM isobutyrate or butyrate.

For HDAC or GPR inhibitors assays, the cells were treated with DMEM containing 2 µM Pimelic Diphenylamide 106 (#19008, MedChem Express LLC, Shanghai, China), 1 µM CHDI‐390576 (#109707, MedChem Express LLC, Shanghai, China), 1 µM Tubastatin A (#313908, MedChem Express LLC, Shanghai, China), 25 µM SIS17 (#272897, MedChem Express LLC, Shanghai, China), 100 nM BRD‐6929 (#60769, MedChem Express LLC, Shanghai, China), 100 nM Santacruzamate A (#17907, MedChem Express LLC, Shanghai, China), 1 µM PCI‐34051 (#22985, MedChem Express LLC, Shanghai, China), 10 µM RGFP966 (#336912, MedChem Express LLC, Shanghai, China), 1 µM GLPG0974 (#90520, MedChem Express LLC, Shanghai, China), 1 µM Mepenzolate (#420181, MedChem Express LLC, Shanghai, China).

### Fecal DNA Extraction and Quantification of Bacteria

DNA was extracted by a fecal genome DNA extraction kit (#DP328‐02, TIANGEN, Beijing, China) according to the manufacturer's protocol. Q‐PCR assays were performed by the ChamQ SYBR qPCR Master Mix (Q331‐02, Vazyme Biotech Co.,Ltd, Nanjing, China). The primer used to amplify the bacteria was described in Table S2 (Supporting Information).

### 16S rDNA Sequencing

Genomic DNA was extracted from fecal samples using a fecal genome DNA extraction kit. The primers 338F and 806R were employed to amplify V3‐V4 area of the 16S rRNA gene. The sequencing service was provided by Beijing Biomarker Technologies Co., Ltd. (Beijing, China). Illumina NovaSeq 6000 (San Diego, California, USA) sequencing platform were conducted. The QIIME2 data analysis package was used for 16S rDNA data analysis. To classify and organize the obtained sequences, the DADA2 clustering program was applied. This program grouped the sequences into operational taxonomic units (OTUs) by comparing them against the Greengene database, with a pre – set criterion of 100% amplicon sequence variant (ASV) identity. Data were analyzed on the free online platform BMKCloud (www.biocloud.net).

### Statistical Analysis

All statistical analyses were performed using GraphPad Prism software. ImageJ was used for the quantifications of histopathological staining. Data were shown as means ± SD. Multi group comparisons were performed using one‐way ANOVA (Dunnett‐t test) or the Kruskal‐Wallis test. Student's t‐test or Mann‐Whitney U test was used for comparisons between two groups. At least three independent experiments were performed.

## Conflict of Interest

The authors declare no conflict of interest.

## Author Contributions

Yimeng Chen and Shujun Jiang contributed equally to this work. Yimeng Chen, Xiaodong Wen and Jie Yang designed the project and wrote the manuscript. Yimeng Chen, Shujun Jiang and Haolu Wang performed experiments and interpreted the data. Mengzhen Si, Yimeng Chen, Shujun Jiang, Haixia Wu and Yanliang Zhang recruited subjects and collected samples. Xinyi Liang and Shenmeng Yao perfomed formal analysis and investigated. Xiaodong Wen and Jie Yang supervised the project and funded the project.

## Supporting information



Supporting Information

## Data Availability

The authors confirm that the data supporting the findings of this study are available within the article and its supplementary materials. 16S sequencing and RNA‐seq data have been deposited at SRA (PRJNA1215536) and GEO database (GSE288546).
