# Supplementary material for: The Probiotic Parabacteroides johnsonii Ameliorates Metabolic Disorders Through Promoting BCAAs to BSCFAs Conversion
Source: Adv Sci (Weinh). 2025 Aug 7;12(38):e02624. doi: 10.1002/advs.202502624 (PMC12520484; doi:10.1002/advs.202502624)
Supplement: Supplementary file 1 — Supporting Information [file ADVS-12-e02624-s001.docx]

## Supplementary materials

**Supplementary tables**

**Table S1** Characteristics of the population and general confounders

| **Table S1** Characteristics of the population and general confounders | | | |
| --- | --- | --- | --- |
| Parameter | Control (n=39) | Obesity (n=42) | *p*-value |
| Gender (F/M) | 11/28 | 12/30 |  |
| Age (year) | 35.6±7.6 | 35.6±10.6 | 0.7 |
| BMI (kg/m^2^) | 22.4±1.6 | 31.2±4.4 | <0.0001 |
| FBG (mmol/L) | 5.1±0.4 | 5.9±0.8 | <0.0001 |
| TG (mmol/L) | 1.3±0.9 | 2.8±1.9 | <0.0001 |
| TC (mmol/L) | 4.9±0.9 | 5.7±1.4 | 0.0067 |
| LDL-C (mmol/L) | 2.9±0.7 | 3.3±0.9 | 0.0415 |
| HDL-C (mmol/L) | 1.4±0.3 | 1.2±0.2 | 0.0042 |
| HbA1c (%) | 5.2±0.3 | 5.6±0.5 | 0.0003 |

**Table S2** List of all primer sequences used in Q-PCR and cut & run assay

| Experiment | Gene | Sequence (5' to 3') |
| --- | --- | --- |
| Q-PCR for mRNA level  in mouse | *Fgf1a* | F: CCCAAAGCCAAGAAGCCACC |
|  |  | R: TGTGCTGGTCGCTCCTGTCCCT |
|  | *Fgf1b* | F: TCAGTCCAGGCACCCTGT |
|  |  | R: GGGGCTCTCTTCACTCCACT |
|  | *Fgf1g* | F: TGATCTGAAGACAAACGAGCA |
|  |  | R: TCCTAGAGATTCCTGCCCTTC |
|  | *Il6* | F: CACGGCCTTCCCTACTTCAC |
|  |  | R:TGCAAGTGCATCATCGTTGT |
|  | *Il1β* | F: TGGACCTTCCAGGATGAGGACA |
|  |  | R: GTTCATCTCGGAGCCTGTAGTG |
|  | *Mcp1* | F: GCTGGAGAGCTACAAGAGGATC |
|  |  | R: GTCAACTTCACATTCAAAGTGC |
|  | *β-actin* | F: CATTGCTGACAGGATGCAGAAGG |
|  |  | R: TGCTGGAAGGTGGACAGTGAGG |
|  | *Tjp1* | F: GCCGCTAAGAGCACAGCAA |
|  |  | R: TCCCCACTCTGAAAATGAGGA |
|  | *Oclludin* | F: TTGAAAGTCCACCTCCTTACAGA |
|  |  | R: CCGGATAAAAAGAGTACGCTGG |
|  | *Fgf1* | F: CCCTGACCGAGAGGTTCAAC |
|  |  | R: GTCCCTTGTCCCATCCACG |
|  | *Fasn* | F: CACAGTGCTCAAAGGACATGCC  R: CACCAGGTGTAGTGCCTTCCTC |
|  | *Srebp1* | F: CGACTACATCCGCTTCTTGCAG |
|  |  | R: CCTCCATAGACACATCTGTGCC |

Continue. **Table S2**

| Experiment | Gene | | Sequence (5' to 3') |  |
| --- | --- | --- | --- | --- |
| Q-PCR for mRNA level  in mouse | *Pgc1a* | | F: ACAACCGCAGTCGCAACA |  |
|  |  |  | R: GGAGGAGTCGTGGGAGGAG |  |
|  | *Cpt1a* | | F: TAGGACAGGCAGAAAATTGT |  |
|  |  |  | R: CATTAGGAGCCGATTCAAAA |  |
|  | *Fabp1* | | F: GTGACTGAACTCAATGGAGACAC |  |
|  |  |  | R: GTAGACAATGTCGCCCAATGTCA |  |
|  | *Fatp2* | | F: GCTGACATCGTAGGACTGGT |  |
|  |  |  | R: TTCGACCCTCATGACCTGGC |  |
|  | *Cd36* | | F: ATGGGCTGTGATCGGAACTG |  |
|  |  |  | R: TTTGCCACGTCATCTGGGTTT |  |
|  | *Apob48* | | F: TGGGATTCCATCTGCCATCTCGAG |  |
|  |  | | R: GTAGAGATCCATCACAGGATAATG |  |
|  | *Fatp4* | | F: AGCAACACCAGGCTTAGGCC |  |
|  |  | | R: TGCTCTTGTCTGTGTGAATG |  |
|  | *Dgat* | | F: GTGCACAAGTGGTGCATCAG |  |
|  |  | | R: CAGTGGGACCTGAGCCATCA |  |
|  | *Lpcat3* | | F: CTACCCGTTGGCTCTGTTTTAC |  |
|  |  | | R: TGAAGCACGACACATAGCAAG |  |
| Q-PCR for mRNA level  in NCM460 cells | | *FGF1A* | F: AGTGGATCCAACAGCCTTCG | |
|  |  | *FGF1B* | F: CCTCGGCCTACAAGCTCTTT | |
|  |  | *FGF1C* | F: CTCAAAGAAGGGCTTTGCCAC | |
|  |  | *FGF1D* | F: CTGTTGGCAGCAGCACAATG | |
|  |  | *FGF1* | R: CTGTCCCTTGTCCCATCCAC | |
|  |  | *IL6* | F: AGACAGCCACTCACCTCTTCAG | |
|  |  |  | R: TTCTGCCAGTGCCTCTTTGCTG | |
|  |  | *IL1β* | F: ATGGCTTATTACAGTGGCA | |
|  |  |  | R: TGTAGTGGTGGTCGGAGA | |
|  |  | *MCP1* | F: CAGCCAGATGCAATCAATGCC | |
|  |  |  | R: TGGAATCCTGAACCCACTTCT | |
|  |  | *TJP1* | F: GTCCAGAATCTCGGAAAAGTGCC | |
|  |  |  | R: CTTTCAGCGCACCATACCAACC | |
|  |  | *OCLN* | F: ATGGCAAAGTGAATGACAAGCGG | |
|  |  |  | R:CTGTAACGAGGCTGCCTGAAGT | |
|  |  | *GAPDH* | F: GTCTCCTCTGACTTCAACAGCG | |
|  |  |  | R: ACCACCCTGTTGCTGTAGCCAA | |
| Q-PCR for cut& run assay  in NCM460 cells | | *FGF1B* | F: GCAGGGATGCCAGATGACA | |
|  |  |  | F: TGTGTGAGCCGAATGGACTTC | |

Continue. **Table S2**

| Experiment | Gene | Sequence (5' to 3') |
| --- | --- | --- |
| Q-PCR for *Parabacteroides* | *P. distasonis* | F: TGCCTATCAGAGGGGGATAAC |
|  |  | R: GCAAATATTCCCATGCGGGAT |
|  | *P. goldsteinii* | F: GAATAAAGTGAGGAACGTGTT |
|  |  | R: AACTTTCACCGCTGACTTAATTA |
|  | *P. merdae* | F: AGTGTGTTTGAGGTAGGCGG |
|  |  | R: ACGCTTTCGCTGTAGAGCTT |
|  | *P. johnsonii* | F: CCTTGAAAGAGGTCTTCTAGCAATAGCT |
|  |  | R: GACCTGTTAGTAACTAGTGAT |
|  | *universal Eubacteria* | F: CGGCAACGAGCGCAACCC |
|  |  | R: CCATTGTAGCACGTGTGTAGCC |

**Supplementary figures**


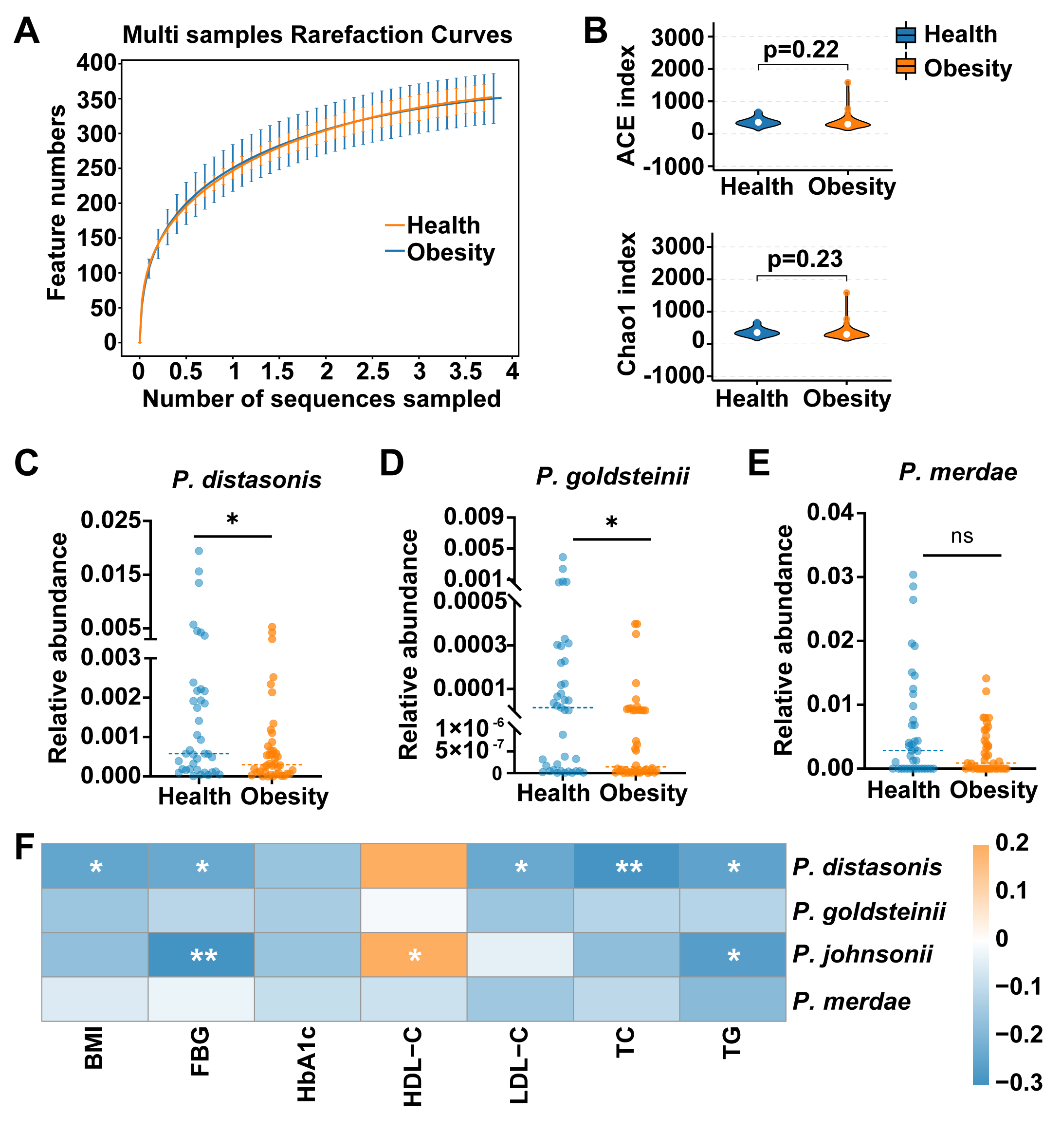


**Figure S1.** Gut microbiota profiling in obese patients. (A) Rarefaction curve. (B) Alpha diversity analysis. Relative abundance of *P. distasonis* (C), *P. goldsteinii* (D), and *P. merdae* (E), analyzed by Q-PCR. (F) Spearman’s analysis of the correlations between the relative abundance of microbiota and blood biochemical indexes in patients levels. Health, healthy subjects (n=39); Obesity, obese patients (n=42); (B - E) values are means±SD, analyzed by Mann-Whitney test. Health *vs.* Obesity, *^*^p*<0.05; ns, no significant difference.


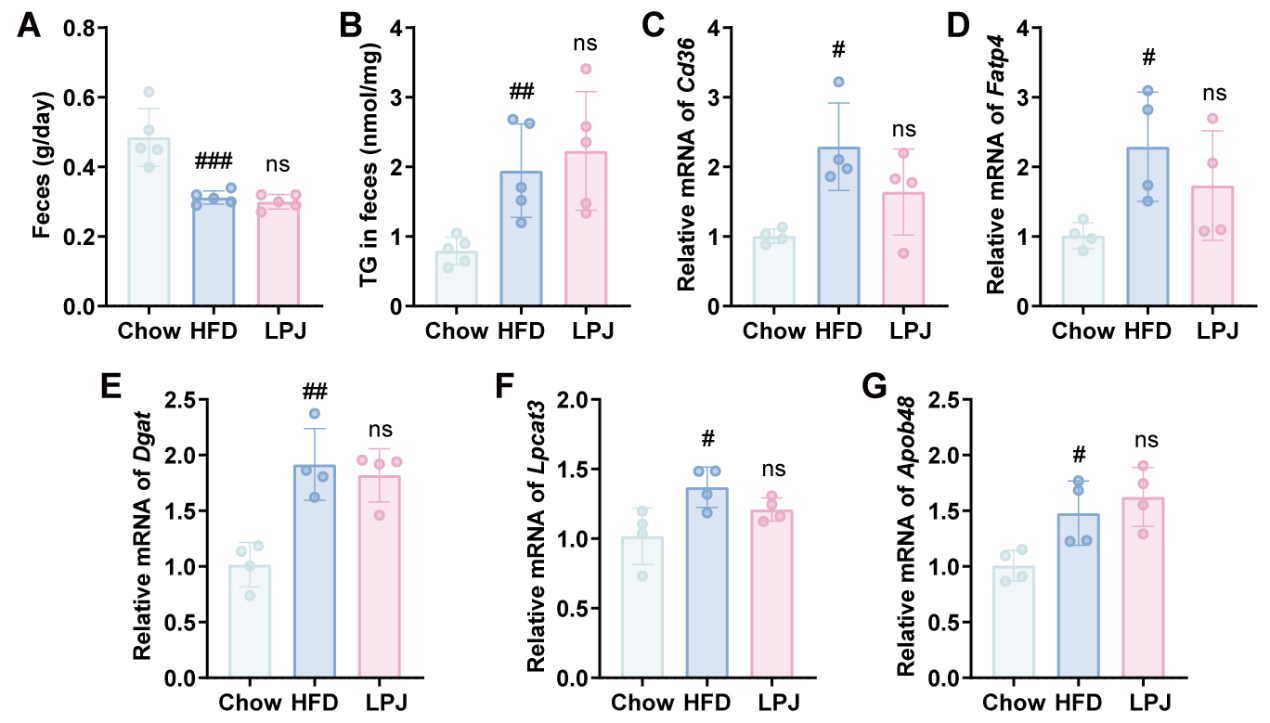


**Figure S2.** Effects of LPJ on jejunal lipid metabolism. (A) The fecal excretion volume. (B) The content of TG in feces. (C-G) The change of genes related to lipid metabolism in the jejunum. Relative mRNA of *Cd36* (C), *Fatp4* (D), *Dgat* (E), *Lpcat3* (F), *Apob48* (G). Chow, chow diet-fed mice; HFD, high fat diet-fed mice; LPJ, live *P. johnsonii* treated mice (n=4-5). Data are presented as the means±SD, analyzed by one-way ANOVA. Chow *vs.* HFD, ^#^*p*<0.05, ^##^*p*<0.01, ^###^*p*<0.001; LPJ *vs.* HFD, ns, no significant difference.


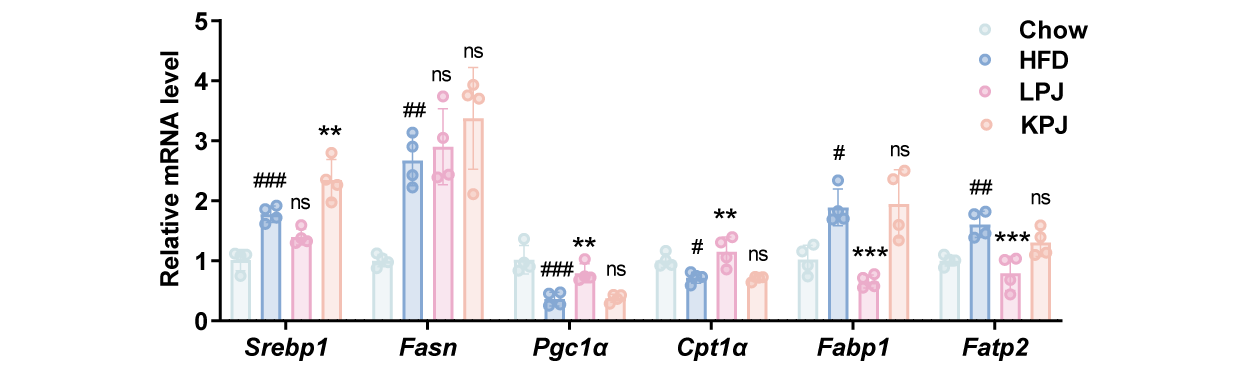


**Figure S3.** The expression of lipid metabolism genes in liver. Chow, chow diet-fed mice; HFD, high fat diet-fed mice; LPJ, live *P. johnsonii* treated mice; KPJ, heat-killed *P. johnsonii* treated mice (n=4). Data are presented as the means±SD, analyzed by one-way ANOVA for 3 or more groups. Chow *vs.* HFD, ^#^*p*<0.05, ^##^*p*<0.01, ^###^*p*<0.001; LPJ or KPJ *vs.* HFD, ^*^*p*<0.05, ^**^*p*<0.01, ^***^*p*<0.001; ns, no significant difference.


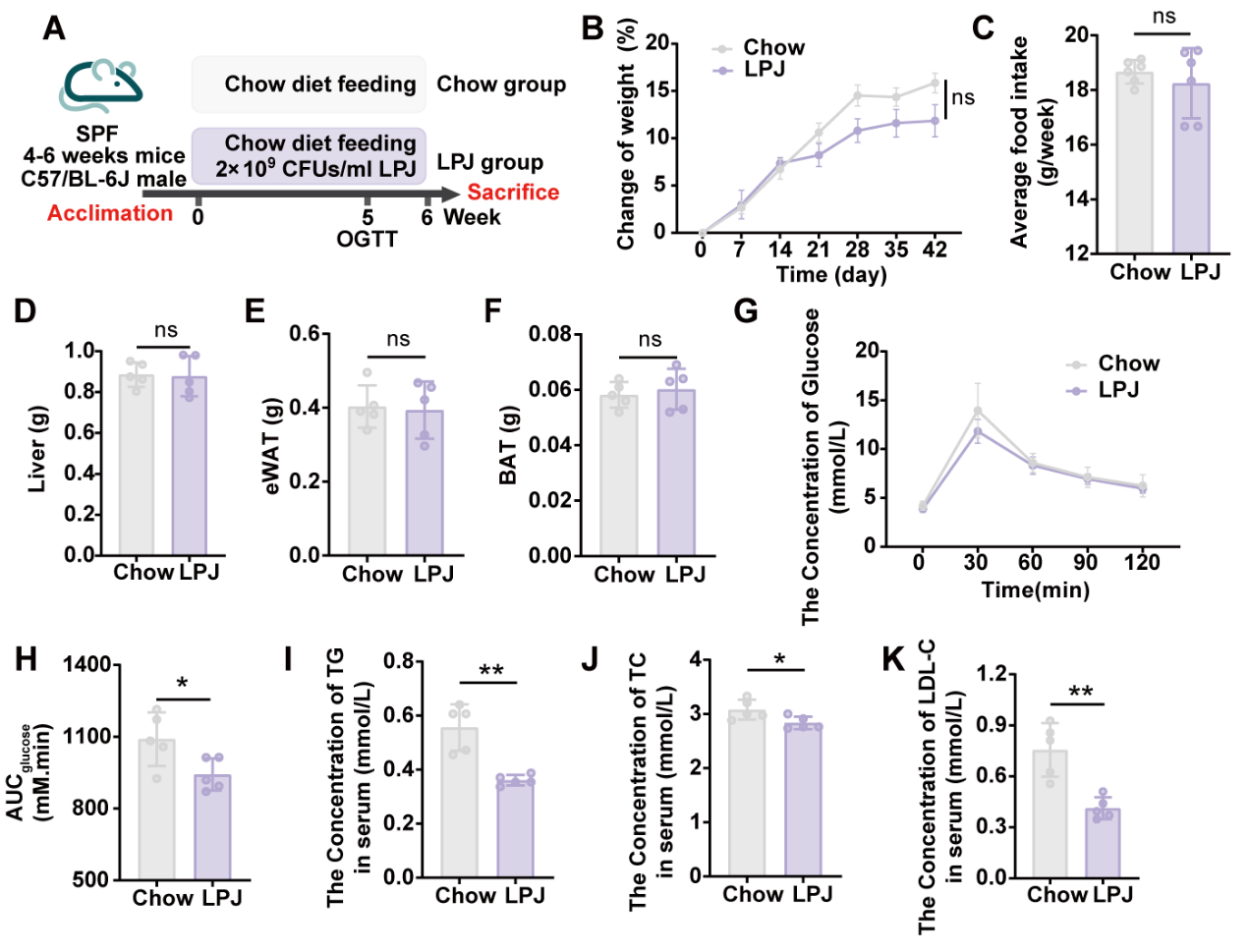


**Figure S4.** The effects of *P. johnsonii* on metabolism of normal mice. (A) Scheme of the experimental design. (B) Body weight change curve. (C) Cumulative food intake. (D - F) The weight of liver (D), eWAT (E), and BAT (F). (G) Oral glucose tolerance test. (H) Area under the curve of OGTT. (I - K) The concentration of TG (I), TC (J), LDL-C (K) in serum. Chow, chow diet-fed mice; LPJ, live *P. johnsonii* treated chow diet-fed mice (n=5). Data are presented as the means±SD, analyzed by unpaired t test. Chow *vs.* LPJ, *^*^p*<0.05, *^**^p*<0.01, *^***^p*<0.001; ns, no significant difference.


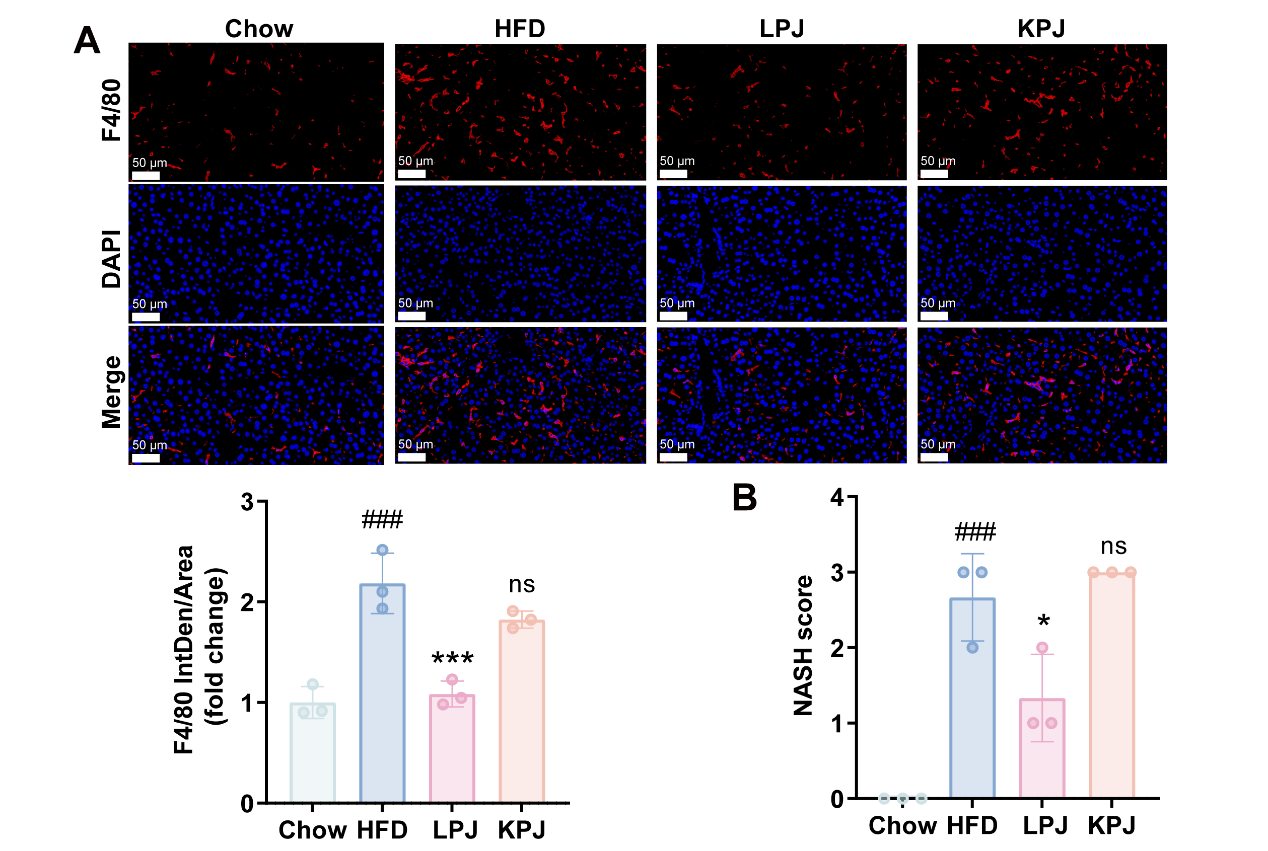


**Figure S5.** F4/80 immunofluorescence staining in liver and NASH score. (A) F4/80 immunofluorescence staining. Scale bars, 50 μm. (B) NASH score. n=3. Chow, chow diet-fed mice; HFD, high fat diet-fed mice; LPJ, live *P. johnsonii* treated mice; KPJ, heat-killed *P. johnsonii* treated mice Data are presented as the means±SD, analyzed by one-way ANOVA. Chow *vs.* HFD, ^###^*p*<0.001; LPJ *vs.* HFD, ^*^*p*<0.05, ^***^*p*<0.001, KPJ *vs.* HFD, ns, no significant difference.


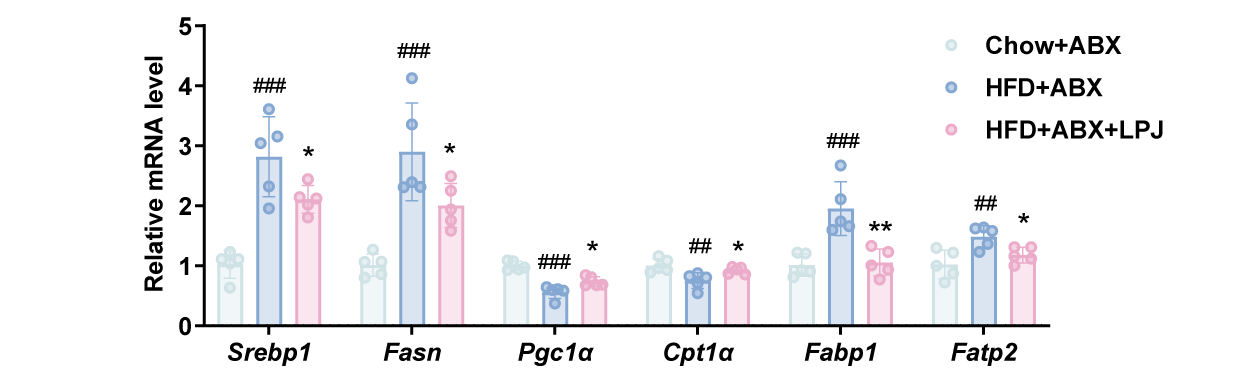


**Figure S6.** The expression of lipid metabolism genes in liver of ABX-HFD mice. ABX, antibiotics; Chow, chow diet-fed mice; HFD, high fat diet-fed mice; LPJ, live *P. johnsonii* (n=5). Data are presented as the means±SD, analyzed by one-way ANOVA. Chow+ABX *vs.* HFD+ABX, ^#^*p*<0.05, ^##^*p*<0.01, ^###^*p*<0.001; HFD+ABX+LPJ *vs.* HFD+ABX, ^*^*p*<0.05, ^**^*p*<0.01, ^***^*p*<0.001, ns, no significant difference.


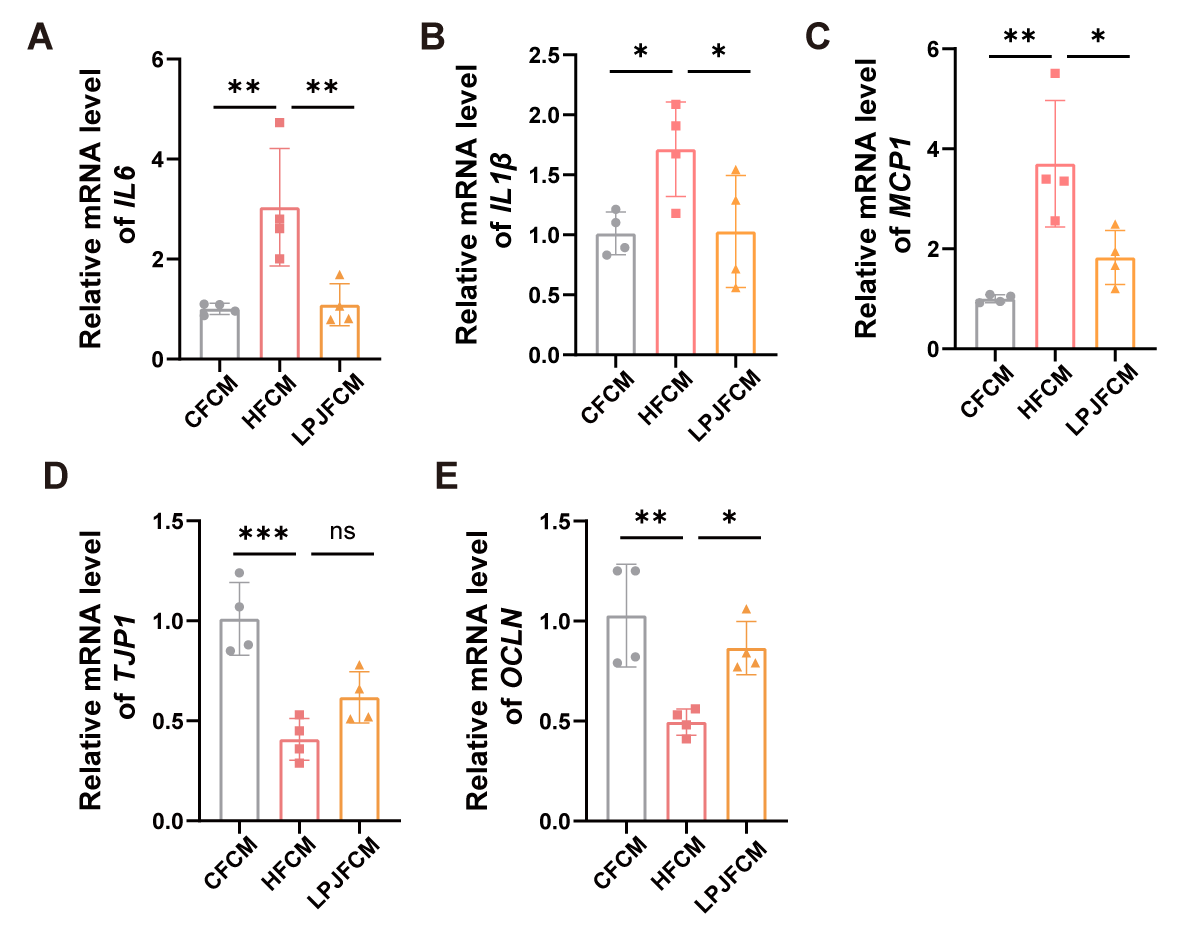


**Figure S7.** Effects of fecal supernatant from ABX treated mice on the mRNA expressions of inflammatory factors and tight junction protein in NCM460 cells. (A) The expression of *IL6* in NCM460. (B) The expression of *IL1β* in NCM460. (C) The expression of *MCP1* in NCM460. (D) The expression of *TJP1* in NCM460. (E) The expression of *OCLN* in NCM460. CFCM, Fecal conditioned media from chow+ABX group; HFCM, Fecal conditioned media from HFD+ABX group; LPJFCM, Fecal conditioned media from HFD+ABX +LPJ group (n = 4). Data are presented as the means±SD, analyzed by one-way ANOVA. *^*^p*<0.05, *^**^p*<0.01, *^***^p*<0.001, ns, no significant difference.


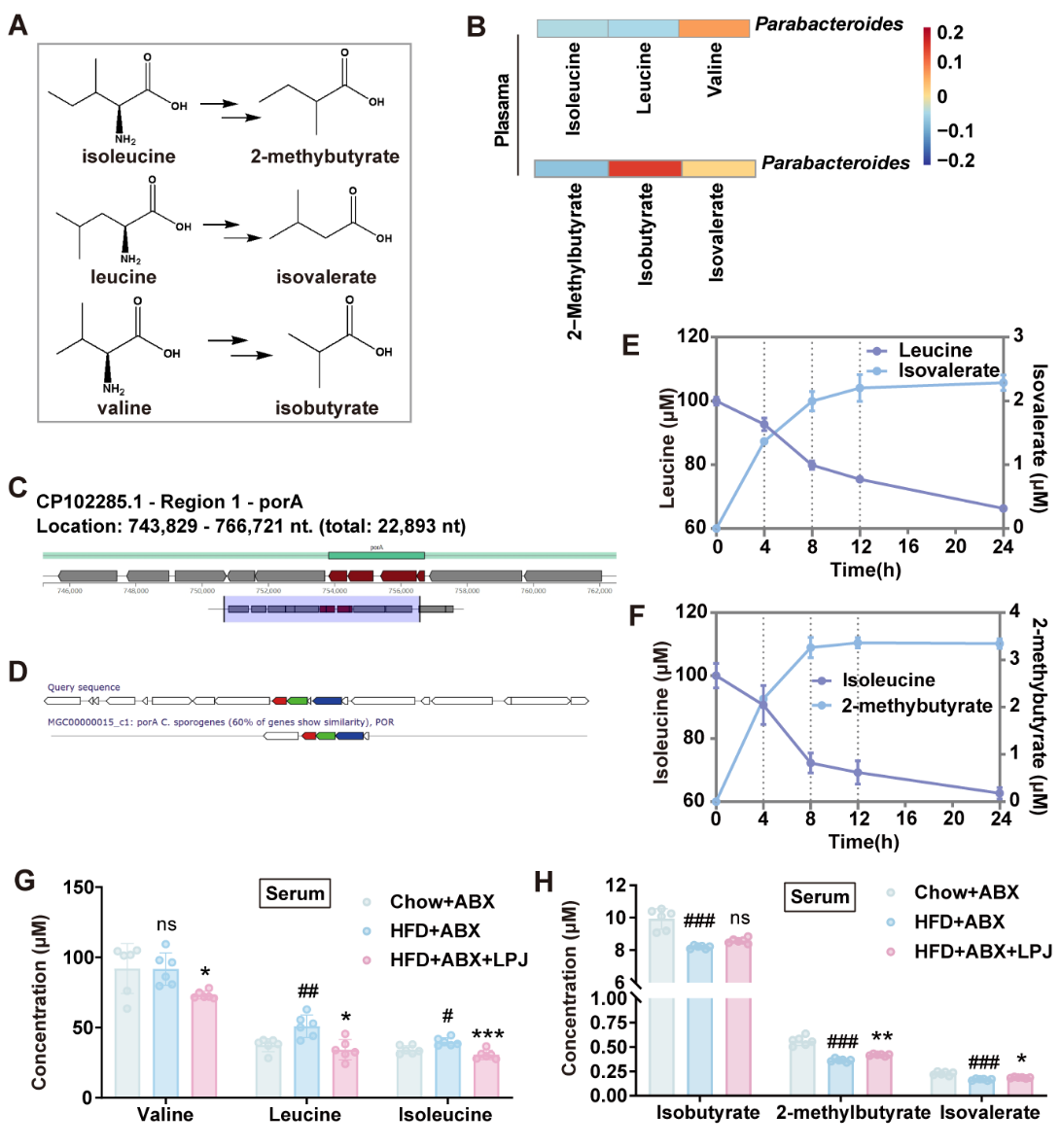


**Figure S8.** The metabolism of BCAAs to BSCFAs by *Parabacteroides*. (A) BCAAs metabolic pathway in the gut. (B) Correlation analysis between *Parabacteroides* and BCAAs or BSCFAs in plasma. (C) *PorA* gene cluster for BCAAs metabolism predicted by gutSMASH in *P. johnsonii* DSM18315 genome. (D) *PorA* gene cluster in *P. johnsonii* DSM18315 genome and its homologs-MGC00000015_c1 in *Clostridium sporogenes*. (E) Metabolism of leucine by *P. johnsonii* *in vitro* (n = 3). (F) Metabolism of isoleucine by *P. johnsonii* *in vitro* (n = 3). (G) The levels of BCAAs in the serum of pseudo-germ-free mice (n = 6). (H) The levels of BSCFAs in the serum of pseudo-germ-free mice (n = 6). Data are presented as the means±SD, (G, H) analyzed by one-way ANOVA. Chow+ABX *vs.* HFD+ABX, *^#^p*<0.05, *^##^p*<0.01, *^###^p*<0.001; HFD+ABX *vs.* HFD+ABX+LPJ, *^*^p*<0.05, *^**^p*<0.01, *^***^p*<0.001; ns, no significant difference.


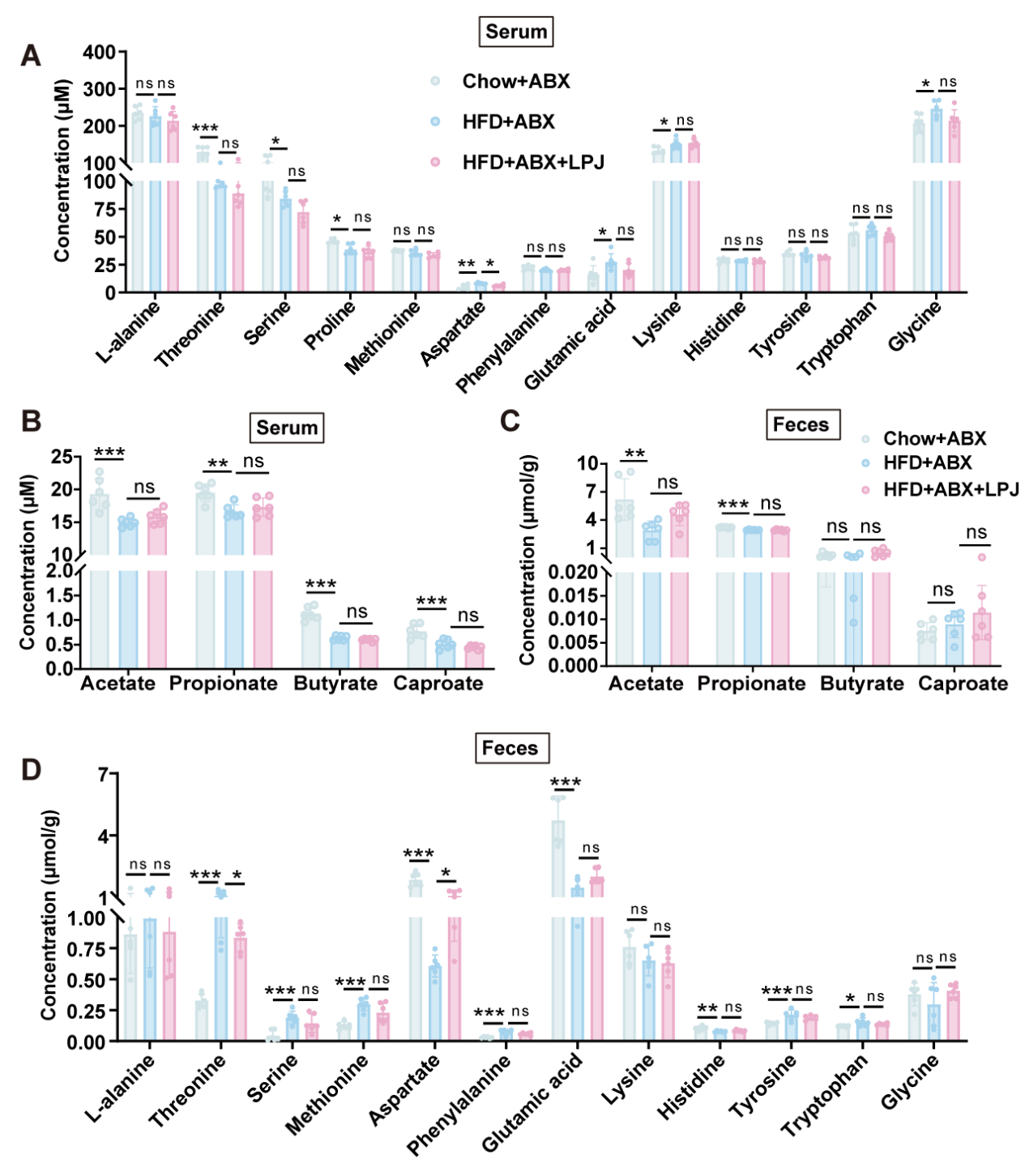


**Figure S9.** Effects of LPJ on metabolism of amino acids and short-chain fatty acids in of pseudo-germ-free mice. (A) The levels of amino acids in serum. (B) The levels of short-chain fatty acids in serum. (C) The levels of short-chain fatty acids in feces. (D) The levels of amino acids in feces. ABX, antibiotics; Chow, chow diet-fed mice; HFD, high fat diet-fed mice; LPJ, live *P. johnsonii* (n=6). Data are presented as the means±SD, analyzed by one-way ANOVA. *^*^p*<0.05, *^**^p*<0.01, *^***^p*<0.001; ns, no significant difference.


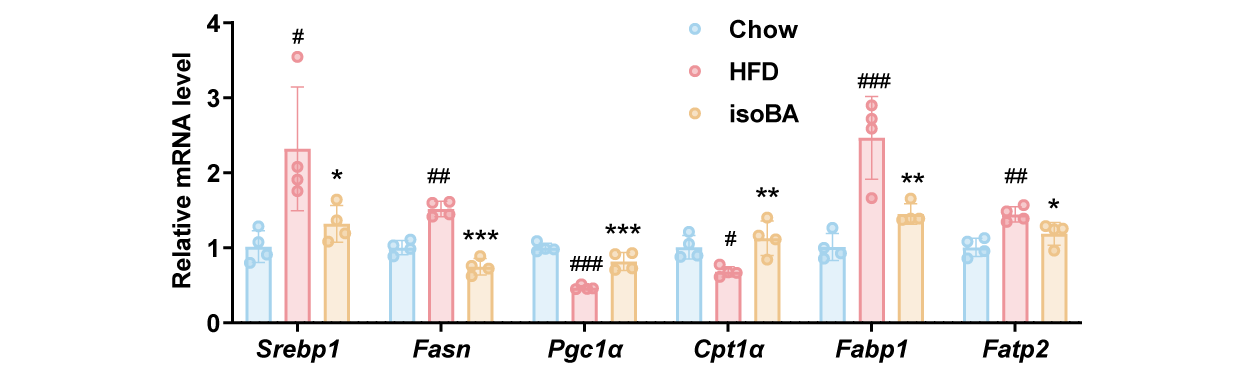


**Figure S10.** The expression of lipid metabolism genes in liver of sodium isobutyrate treated mice. Chow, chow diet-fed mice; HFD, high fat diet-fed mice; isoBA, sodium isobutyrate treated mice (n=4). Data are presented as the means±SD, analyzed by one-way ANOVA. Chow *vs.* HFD, ^#^*p*<0.05, ^##^*p*<0.01, ^###^*p*<0.001; isoBA *vs.* HFD, ^*^*p*<0.05, ^**^*p*<0.01, ^***^*p*<0.001, ns, no significant difference.


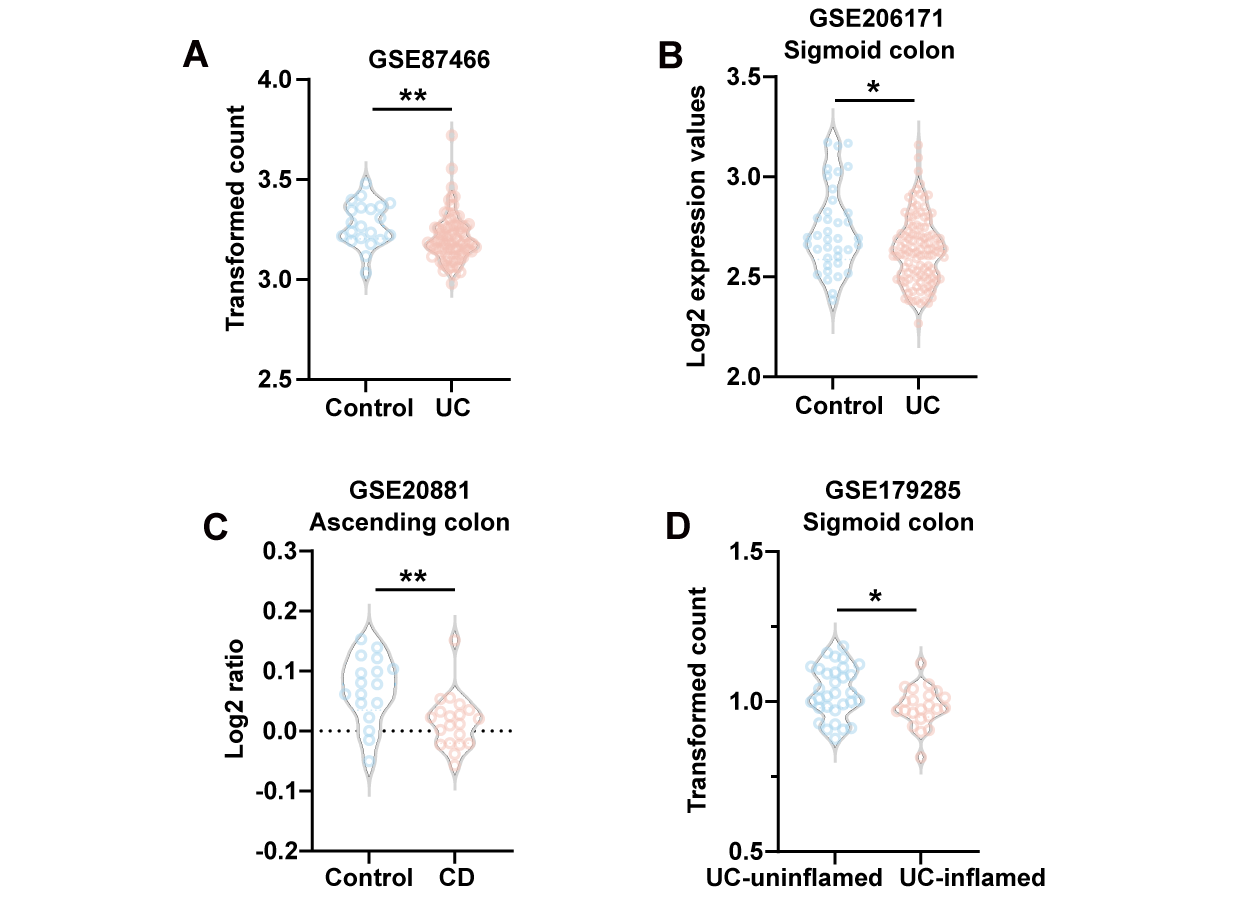


**Figure S11.** Expression of *FGF1* in tissues of UC or CD patients as revealed by four independent GEO data sets. (A-B) Expression of *FGF1* in tissues of UC. (C) Expression of *FGF1* in tissues of CD patients. (D) Expression of *FGF1* in tissues of UC with inflammatory or non-inflammatory state. GSE87466 (Control, n = 21; UC, n = 87), GSE206171 (Control, n = 38; UC, n = 114), GSE20881 (Control, n = 17; CD, n = 20), GSE179285 (UC-uninflamed, n = 31; UC-inflamed, n = 22). Analyzed by Mann-Whitney test. ^*^p<0.05, ^**^p<0.01.


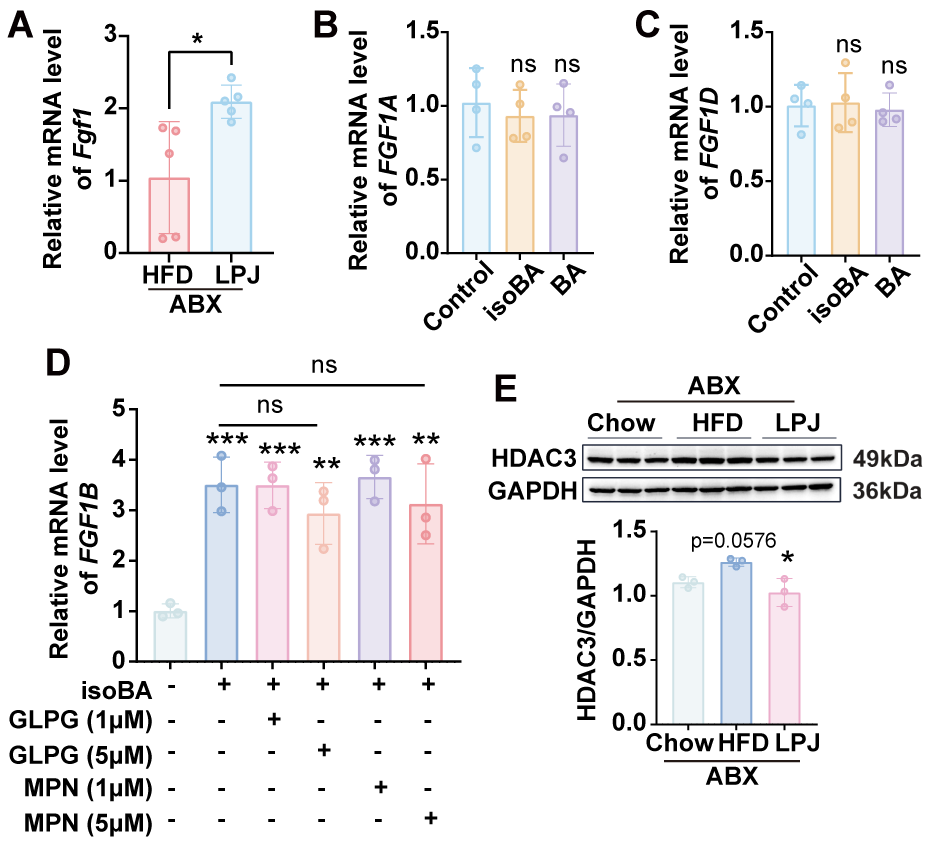


**Figure S12.** Effects of LPJ or isobutyrate on FGF1 expression *in vivo* and *in vitro*. (A) Effect LPJ on *Fgf1* expression in colon of HFD-fed pseudo-germ-free mice (n = 5). (B) Effect of isobutyrate on *FGF1A* expression in NCM460 cells (n=4)*.* (C) Effect of isobutyrte on *FGF1D* expression in NCM460 cells (n=4)*.* (D) Effect of GPCR43 antagonist or GPCR109A antagonist on isobutyrate*-*induced *FGF1B* expression in NCM460 cells (n = 3). (E) The expression of HDAC3 in the colon of HFD-fed pseudo-germ-free mice (n = 3). Chow, chow diet-fed mice; HFD, high fat diet-fed mice; LPJ, live *P. johnsonii*; ABX, antibiotics; GLPG, GLPG0974; MPN, Mepenzolate bromide. Data are presented as the means±SD, analyzed by unpaired t test in (A) and by one-way ANOVA in (B-E). Other groups *vs.* Control or LPJ *vs.* HFD, *^*^p*<0.05, *^**^p*<0.01, *^***^p*<0.001, ns, no significant difference.


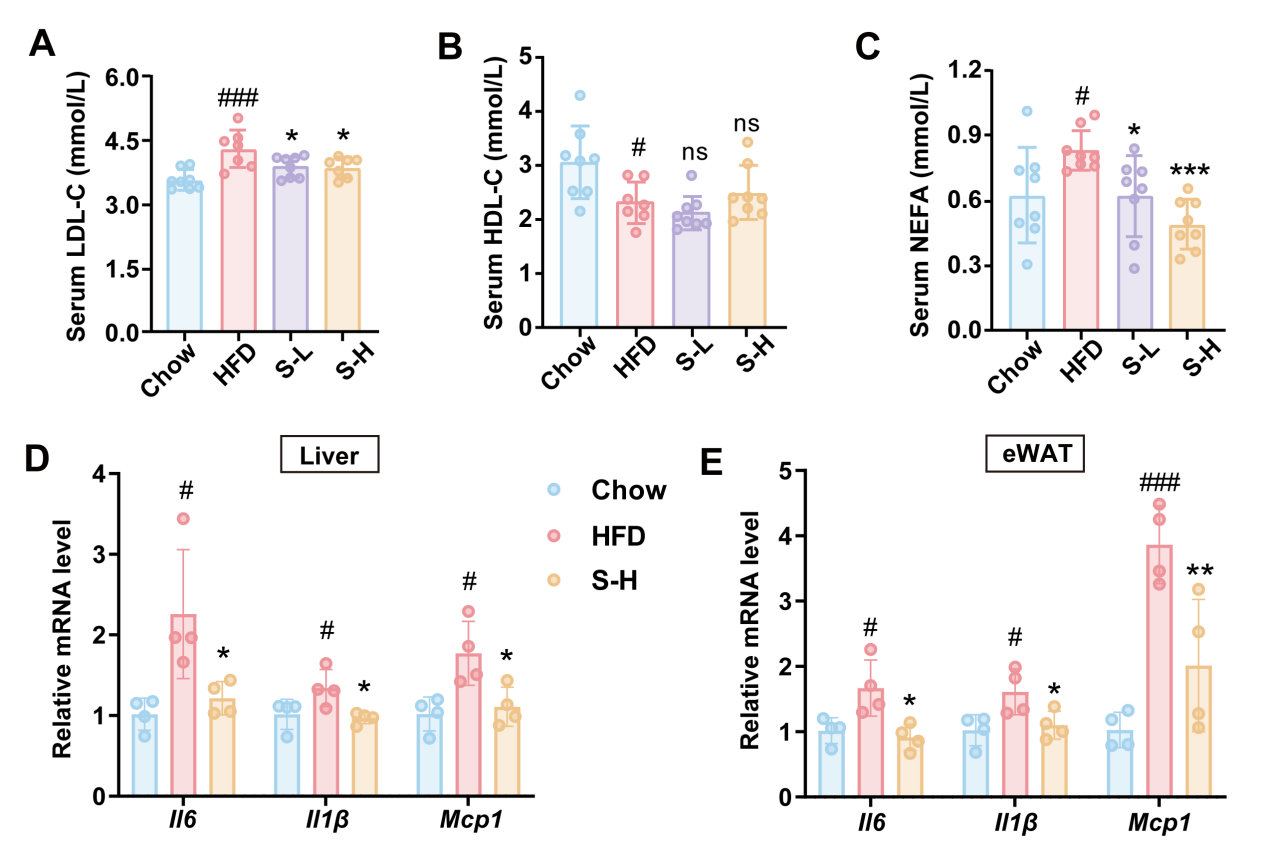


**Figure S13.** Effects of stachyose on metabolism and inflammation in HFD-fed mice. (A - C) The concentration of LDL-C (A), HDL-C (B), and NEFA (C). (D) Relative mRNA levels of inflammatory factors in liver. (E) Relative mRNA levels of inflammatory factors in WAT. Chow, chow diet-fed mice; HFD, high fat diet-fed mice; S-L, low dose of stachyose treated HFD-fed mice; S-H, high dose of stachyose treated HFD-fed mice, n = 4 - 8. Data are presented as the means±SD, analyzed by one-way ANOVA. Chow *vs.* HFD, *^#^p*<0.05, *^##^p*<0.01, *^###^p*<0.001; S-L or S-H *vs.* HFD, *^*^p*<0.05, *^**^p*<0.01, *^***^p*<0.001; ns, no significant difference.


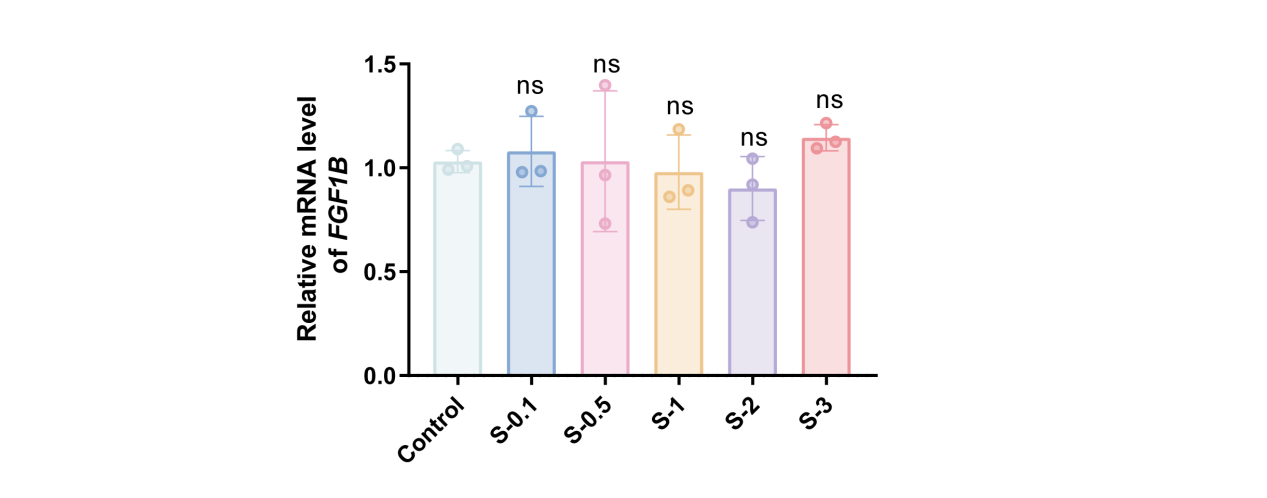


**Figure S14.** Effects of stachyose on the expression of *FGF1B* in NCM460 cells. S-0.1, 0.1 mg/ml stachyose, S-0.5, 0.5 mg/ml stachyose, S-1, 1 mg/ml stachyose, S-2, 2 mg/ml stachyose, S-3, 3 mg/ml stachyose, n = 3. Data are presented as the means±SD, analyzed by one-way ANOVA. Stachyose *vs.* Control, ns, no significant difference.

**Supplementary methods**

**Culture and Preparation of *P. johnsonii***

*P. johnsonii* (strain: DSM18315) was purchased from the German Collection of Microorganisms and cultivated in YCFA medium (containing of 1 g casitone, 0.5 g glucose, 0.25 g yeast extract, 0.4 g NaHCO_3_, 0.1 g cysteine, 0.045 g K_2_HPO_4_, 0.045 g KH_2_PO_4_, 0.09 g NaCl, 0.09 g MgSO_4_.7H_2_O, 0.09 g CaCl_2_ ,0.1 mg resazurin, 1.5 mg haemin, 0.1 mg vitamin K1, 1 µg biotin, 1 µg cobalamin, 3 µg p-aminobenzoic acid, 5 µg folic acid and 15 µg pyridoxamine, per 100 ml). All anaerobic microbiological experiments were carried out in an anaerobic chamber filled with a gas mixture of 10% CO_2_, 10% H_2_, and 80% N_2_. The cultures were harvested during the log phase. They were then diluted to a concentration of 2 × 10^9^ CFUs per milliliter using sterile phosphate - buffered saline (PBS) for gavage. For the heat-killed *P. johnsonii* (KPJ) trials, *P. johnsonii* were heat-killed at 121 ℃ for 20 min.

***P. johnsonii* growth assay in vitro**

The cultivated *P. johnsonii* was cultured in YCFA without glucose, and seeded in 96-well plates under the initial OD600 value of 0.1. *P. johnsonii* was treated with 0.5% glucose or 0.5% stachyose. Then, the OD600 value was recorded after co-cultivation at plateau.

**Oral glucose tolerance test (OGTT)**

The mice which fasted overnight were orally administered a glucose solution at a dose of 2 g/kg body weight.^[1]^ Blood samples were then collected from the tail vein and glucose levels were sequentially measured at 0, 30, 60, 120, and 180 minutes using a glucometer (Sannuo, Changsha, China).

**Biochemical measurements**

The biochemical measurements of serum (including triglyceride, total cholesterol, low-density lipoprotein cholesterol, high density lipoprotein cholesterol and nonestesterified fatty acid) were assayed by kits according to the manufacturer’s instructions (Jiancheng Bioengineering Research Institute, Nanjing, China). Lipopolysaccharide (LPS) was determined by ELISA kits (AiFang biological, Changsha, China).

**Gut permeability assays**

FD4 permeability assays were performed as previously described.^[2]^ Briefly, mice were fasted for 6 hours, oral gavage with 400 mg/kg body weight of Fluorescein isothiocyanate dextran 4KDa (FD4, [Sigma-Aldrich](https://www.sigmaaldrich.cn/CN/zh/life-science/sigma-aldrich), Darmstadt, Germany) and then the blood were collected from the saphenous vein at the 1 and 4 h time-points. Serum was separated by centrifuging the samples at 1000×*g* at 4℃ for 10 min. Standards were prepared using plasma spiked with FD4 at varying concentrations. Samples were diluted with PBS 1:4 before loading in a 96-well flat bottom plate and fluorescence was measured at 485/525 nm.

**Immunofluorescence staining**

The colon tissues were fixed in 4% paraformaldehyde (Servicebio, G1101) and embedded by paraffin. For immunofluorescence of ZO-1 and Occludin in the colon tissues, the slides of the tissues were incubated with ZO-1 (sc-33725, Santa Cruz Biotechnology, Santa Cruz, California, USA) and Occludin (ab216327, Abcam, Cambridge, UK) antibody overnight at 4℃ in a humidified dark chamber, and then incubated with Fluorescein (FITC)-conjugated Affinipure Goat Anti-Rabbit IgG (H + L) and Fluorescein (FITC)-conjugated Affinipure Goat Anti-Rat IgG (H + L) for 1 h at room temperature. Images were acquired by confocal laser scanning microscope.

**Histopathological and Immunohistochemical Analysis**

The tissues were fixed in 4% paraformaldehyde (Servicebio, G1101) and embedded by paraffin. The tissues were cut into 5 μm thickness and stained with hematoxylin and eosin. Images were captured using NanoZoomer 2.0 (Hamamatsu, Japan). For immunohistochemistry (IHC), paraffin sections were incubated with antibodies specific to TLR4.

**Metabolomics profiling of serum and fecal samples**

The levels of BSCFAs and BCAAs were determined by LC-MS.^[3]^ For serum of mice or plasma of volunteers, 50 μl of each sample was thoroughly mixed with ice - cold acetonitrile at a volume ratio of 1:3. After centrifugation, 40 μl supernatant was transferred and combined with 20 μl of 175 mM solution of 3-nitrophenylhydrazine (3-NPH, [Sigma-Aldrich](https://www.sigmaaldrich.cn/CN/zh/life-science/sigma-aldrich), Darmstadt, Germany) in 50 % acetonitrile aqueous solution. Additionally, 20 μl of 105 mM 1-(3-Dimethylaminopropyl)-3-ethylcarbodiimide hydrochloride (EDC, [Sigma-Aldrich](https://www.sigmaaldrich.cn/CN/zh/life-science/sigma-aldrich), Darmstadt, Germany) in 2.5 % pyridine in 50 % acetonitrile aqueous solution was added, along with 10 μl of an internal standard containing 200 μM acetic acid - d4 and 50 μM leucine - d7. For feces of mice or volunteers, 50 mg samples were homogenized in deionized water (1:10 w/v), centrifuging to get supernatant. 50 μl fecal supernatant were used for the deproteinization process. Then, 40 μl supernatant solution was mixed with 20 μl of 3-NPH, 20 μl of EDC, and 10 μl internal standard (5 mM acetic acid-d_4_ and 50 μM leucine-d_7_). After reaction at 40℃ for 30 min, the mixtures were filled to 200 μl (serum or plasma samples) or 500 μl (fecal samples) with 10 % acetonitrile solution and centrifuged. AB Sciex Triple Quad™ 4500 system (Applied Biosystems Inc., USA), with a BEH-C_18_ column (2.1 mm×100 mm, 1.7 μm, waters) were used for quantitative analysis and the gradient condition was as previous study ^3^. The collection of data were carried out using Analyst software version 1.7. To precisely monitor and quantify the transitions from precursor ions to product ions, the multiple reaction monitoring (MRM) mode was adopted.

**HDAC activity analysis**

HDAC activity analysis was performed according to the protocol from HDAC Assay Kit (Fluorescent) (56200, Active Motif, Carlsbad, California, USA). Briefly, 45 nM of recombinant protein rHDAC3/NCOR2 (31526, Active Motif, Carlsbad, California, USA) was incubated with 10 μM, 100 μM, 1 mM, 5 mM, and 10 mM isobutyrate or 100 μM, 1 mM, 5 mM, and 10 mM butyrate for 60 min at 37^o^C. 50 μl of developer solution containing 2 μM of trichostatin A (TSA) was added at room temperature to stop the reaction. Fluorescence was measured using a fluorescent plate reader with an excitation wavelength of 340 nm and emission wavelength of 460 nm.

**CUT & RUN**

CUT&RUN assays were carried out following the protocol provided by the Hyperactive pG - MNase CUT&RUN Assay Kit (#HD101, Vazyme Biotech Co., Ltd, Nanjing, China). In brief, NCM460 cells were first dissociated using trypsin, 1×10⁵ cells per sample were selected for the experiment. Immunoprecipitation was performed at room temperature for 2 hours using antibodies against H3K9ac (5B11, Cell Signaling Technology, Danvers, Massachusetts, USA), H3K14ac (D4B9, Cell Signaling Technology, Danvers, Massachusetts, USA), H3K18ac (ab177870, Abcam, Cambridge, UK), H3K23ac (ab177275, Abcam, Cambridge, UK), and HDAC3 (10255 - 1 - AP, Proteintech, Wuhan, China). The pG - MNase enzyme was allowed to bind to the cells and activated by CaCl₂ for 1.5 hours. The Stop Buffer was added and incubated at 37℃ for 20 minutes. The mixture was centrifuged, and the DNA was enriched by columns. The primers specific for the *FGF1B* promoter: forward primer: 5′-GCAGGGATGCCAGATGACA-3′; reverse primer: 5′-TGTGTGAGCCGAATGGACTTC-3′ were used for QPCR. Spike in DNA was added to each sample for calibration. The data were shown by means of 2^−△△CT^ values and normalized to control group.

**Quantitative PCR (QPCR) analysis**

Total RNA was isolated from the samples using TRIzol reagent. The cDNA was generated through reverse transcription. ChamQ SYBR qPCR Master Mix (Vazyme Biotech Co., Ltd, Nanjing, China) was employed for qPCR. The primer sequences were listed in the Supplementary material **Table S2**.

**Western blot analysis**

The proteins from tissues or cells were extracted with RIPA buffer (Beyotime, Shanghai, China). Equal amounts of protein were loaded. After the separation of proteins by SDS - polyacrylamide gel electrophoresis, the proteins were then transferred onto a PVDF membrane. (Millipore, Billerica, MA, USA). The membranes were incubated with primary antibodies [Anti-HDAC3 (81211-1-RR, Proteintech, Wuhan, China), Anti-FGF1 (17400-1-AP, Proteintech), Anti-H3 (17168-1-AP, Proteintech), Anti-H3ac (AB300641, Abcam), Anti-GAPDH (AP0063, Bioworld, Nanjing, China)] overnight at 4℃ after fast blocking western (Life-iLab, Shanghai, China). Subsequently, secondary antibody was incubated at room temperature. The relative expression level of target protein was normalized to GAPDH.

**RNA sequencing**

The sequencing service was provided by Beijing Biomarker Technologies Co., Ltd. (Beijing, China). First, total RNA was extracted from the colon tissues using TRIzol reagents. The quality of the extracted RNA was then precisely assessed using an Agilent 2100 Bioanalyser. Subsequently, sequencing libraries were constructed and sequenced on an Illumina NovaSeq platform accordance with the manufacturer's instructions. The obtained raw reads underwent further processing via BMKCloud online platform (www.biocloud.net). Differential expression analysis of two groups was performed by DESeq2. Genes with an adjusted P-value < 0.01 & Fold Change≥2 found by DESeq2 were assigned as differential genes.

***FGF1* interference assay**

NCM460 cells were transfected small interfering RNA (siRNA) using Lipofectamine 2000 for 6 h. Scrambled siRNA served as the negative control. SiCtrl (Sense (5’-3’) : UUUCUCCGAACGUGUCACGUTT; Antisense (5 '- 3'): ACGUGACACGUUCGGAGAATT) and siFGF1 (Sense (5’-3’): GGGACAGGAGCGACCAGCATT; Antisense (5’-3’): UGCUGGUCGCUCCUGUCCCTT). After 30 h, the cells were treated with medium containing FCM (1:100, FCM: medium) without or with 10 mM isobutyrate for 12 h.

**Reference**

[1] Chen Y, Zhu L, Hu W, Wang Y, Wen X, Yang J. Simiao Wan modulates the gut microbiota and bile acid metabolism during improving type 2 diabetes mellitus in mice. Phytomedicine 2022;104:154264.

[2] Luck H, Khan S, Kim JH, Copeland JK, Revelo XS, Tsai S, et al. Gut-associated IgA(+) immune cells regulate obesity-related insulin resistance. Nat Commun 2019;10:3650.

[3] Si H, Chen Y, Hu D, Yao S, Yang J, Wen X. A graminan type fructan from Achyranthes bidentata prevents the kidney injury in diabetic mice by regulating gut microbiota. Carbohydr Polym 2024;339:122275.
